# Current Awareness on Comparative and Functional Genomics

**DOI:** 10.1002/cfg.354

**Published:** 2004-07


**Comparative and Functional Genomics**
*Comp Funct Genom* 2004; **5**: 448–455
Published online in Wiley InterScience (www.interscience.wiley.com). **DOI:** 10.1002cfg.354

### Current awareness on comparative and functional genomics

CURRENT AWARENESS - USE THE LINKS ONLINE

2745

### 1 Reviews & symposia

2003. Special issue: Genome research on bacteria relevant for agriculture, environment and biotechnology. *J Biotechnol* **106:** (2-3)

2003. Special issue: Genomics and genetic engineering for the meat industry. *Outlook Agr* **32:** (4)

2004. Special issue: The Molecular Biology Database Collection: 2004. *Nucleic Acids Res* **32:** (1)

2004. Special issue: Nutrigenomics. *Nutrition* **20:** (1)

Auffray C, Imbeaud S, Roux-Rouquie M, Hood L. 2003. From functional genomics to systems biology: Concepts and practices (Review). *C R Biol* **326:** (10-11) 879.

Bacher JM, Bull JJ, Ellington AD. 2003. Evolution of phage with chemically ambiguous proteomes - Online art. no. 24. *BMC Evol Biol* **3:** (1) 24.

Bertucci F, Loriod B, Nasser V, Granjeaud S, Tagett R, Braud AC, Viens P, Houlgatte R, Birnbaum D, Nguyen C. 2003. Gene expression profiling of breast carcinomas using nylon DNA arrays. *C R Biol* **326:** (10-11) 1031.

Brunet S, Thibault P, Gagnon E, Kearney P, Bergeron JJM, Desjardins M. 2004. Organelle proteomics: Looking at less to see more. *Drug Discov Today* **9:** (Suppl 2) S8.

Brunner AM, Busov VB, Strauss SH. 2004. Poplar genome sequence: Functional genomics in an ecologically dominant plant species (Review). *Trends Plant Sci* **9:** (1) 49.

Butterfield DA, Boyd-Kimball D, Castegna A. 2003. Proteomics in Alzheimer's disease: Insights into potential mechanisms of neurodegeneration (Review). *J Neurochem* **86:** (6) 1313.

Carpenter AE, Sabatini DM. 2004. Systematic genome-wide screens of gene function (Review). *Nat Rev Genet* **5:** (1) 11.

Carter MG, Piao YL, Dudekula DB, Qian Y, Van Buren V, Sharov AA, Tanaka TS, Martin PR, Bassey UC, Stagg CA *et al*. 2003. The NIA cDNA project in mouse stem cells and early embryos (Review). *C R Biol* **326:** (10-11) 931.

Conrods TP, Zhou M, Petricoin EF, Liotta L, Veenstra TD. 2003. Cancer diagnosis using proteomic patterns. *Expert Rev Mol Diagn* **3:** (4) 411.

De Angelis M, Gobbetti M. 2004. Environmental stess responses in *Lactobacillus*: A review. *Proteomics* **4:** (1) 106.

Doolan DL, Aguiar JC, Weiss WR, Sette A, Felgner PL, Regis DP, Quinones-Casas P, Yates JR, Blair PL, Ritchie TL, Hoffman SL, Carucci DJ. 2003. Utilization of genomic sequence information to develop malaria vaccines (Review). *J Exp Biol* **206:** (21) 3789.

Dyrskjot L. 2003. Classification of bladder cancer by microarray expression profiling: Towards a general clinical use of microarrays in cancer diagnostics. *Expert Rev Mol Diagn* **3:** (5) 635.

Edwards AM, Kus B, Jansen R, Greenbaum D, Greenblatt J, Gerstein M. 2004. Bridging structural biology and genomics: Assessing protein interaction data with known complexes. *Drug Discov Today* **9:** (Suppl 2) S32.

Espina V, Dettloff KA, Cowherd S, Petricoin EF, Lotta LA. 2004. Use of proteomic analysis to monitor responses to biological therapies. *Expert Opin Biol Ther* **4:** (1) 83.

Francois E, Wang DY, Fulthorpe R, Liss SN, Edwards EA. 2003. DNA microarrays for detecting endocrine-disrupting compounds. *Biotechnol Adv* **22:** (1-2) 17.

Fusaro VA, Stone JH. 2003. Mass spectrometry-based proteomics and analyses of serum: A primer for the clinical investigator (Review). *Clin Exp Rheumatol* **21:** (6 Suppl 32) S3.

Gass S, Harris J, Ormandy C, Brisken C. 2003. Using gene expression arrays to elucidate transcriptional profiles underlying prolactin function. *J Mammary Gland Biol Neoplasia* **8:** (3) 269.

Goldstein AM. 2003. Changing paradigms in dermatology: Proteomics: A new approach to skin disease. *Clin Dermatol* **21:** (5) 370.

Gott JM. 2003. Expanding genome capacity *via* RNA editing (Review). *C R Biol* **326:** (10-11) 901.

Gros F. 2003. From the messenger RNA saga to the transcriptome era (Review). *C R Biol* **326:** (10-11) 893.

Haberkorn U, Altmann A, Mier W, Eisenhut M. 2004. Impact of functional genomics and proteomics on radionuclide imaging. *Semin Nucl Med* **34:** (1) 4.

Han ZG, Zhao GP, Chen Z. 2003. Transcriptome study in China. *C R Biol* **326:** (10-11) 949.

Hayashizaki Y. 2003. The RIKEN mouse genomc encyclopedia product (Review). *C R Biol* **326:** (10-11) 923.

Hayes F. 2003. Transposon-based strategies for microbial functional genomics and proteomics. *Annu Rev Genet* **37:** 3.

Hegde PS, White IR, Debouck C. 2003. Interplay of transcriptomics and proteomics. *Curr Opin Biotechnol* **14:** (6) 647.

Hegde PS, White IR, Debouck C. 2004. Interplay of transcriptomics and proteomics. *Drug Discov Today* **9:** (Suppl 2) S53.

Henshall S. 2003. Tissue microarrays. *J Mammary Gland Biol Neoplasia* **8:** (3) 347.

Jacobsen BM, Richer JK, Sartorius CA, Horwitz KB. 2003. Expression profiling of human breast cancers and gene regulation by progesterone

In order to keep subscribers up-to-date with the latest developments in their field, this current awareness service is provided by John Wiley & Sons and contains newly-published material on comparative and functional genomics. Each bibliography is divided into 16 sections. 1 Reviews & symposia; 2 General; 3 Large-scale sequencing and mapping; 4 Evolutionary genomics; 5 Comparative genomics; 6 Pathways, gene families and regulons; 7 Pharmacogenomics; 8 EST, cDNA and other clone resources; 9 Functional genomics; 10 Transcriptomics; 11 Proteomics; 12 Protein structural genomics; 13 Metabolomics; 14 Genomic approaches to development; 15 Technological advances; 16 Bioinformatics. Within each section, articles are listed in alphabetical order with respect to author. If, in the preceding period, no publications are located relevant to any one of these headings, that section will be omitted.

Copyright © 2004 John Wiley & Sons, Ltd.

receptors. *J Mammary Gland Biol Neoplasia* **8:** (3) 257.

Jeffery DA, Bogyo M. 2004. Chemical proteomics and its application to drug discovery. *Drug Discov Today* **9:** (Suppl 2) S19.

Jones AK, Sattelle DB. 2004. Functional genomics of the nicotinic acetylcholine receptor gene family of the nematode, *Caenorhabditis elegans* (Review). *Bioessays* **26:** (1) 39.

Kato K. 2003. Adaptor-tagged competitive PCR: Study of the mammalian nervous system (Review). *C R Biol* **326:** (10-11) 941.

Lee WC, Lee KH. 2003. Applications of affinity chromatography in proteomics (Review). *Anal Biochem* **324:** (1) 1.

Liang M, Cowley AW, Greene AS. 2004. High throughput gene expression profiling: A molecular approach to integrative physiology. *J Physiol* **554:** (1) 22.

Lijavetzky D, Carbonero P, Vicente-Carbajosa J. 2003. Genome-wide comparative phylogenetic analysis of the rice and *Arabidopsis* Dof gene families - Online art. no. 17. *BMC Evol Biol* **3:** (1) 17.

Liu Y, Sturley SL. 2004. Nutritional genomics in yeast models. *Nutrition* **20:** (1) 166.

Lock CB, Heller RA. 2003. Gene microarray analysis of multiple sclerosis lesions (Review). *Trends Mol Med* **9:** (12) 535.

McGrath CL, Katz LA. 2004. Genome diversity in microbial eukaryotes (Review). *Trends Ecol Evol* **19:** (1) 32.

McShane LM, Shih JH, Michalowska AM. 2003. Statistical issues in the design and analysis of gene expression microarray studies of animal models. *J Mammary Gland Biol Neoplasia* **8:** (3) 359.

Meng ZJ, Simmons-Willis TA, Limbach PA. 2004. The use of mass spectrometry in genomics (Review). *Biomol Eng* **21:** (1) 1.

Minamikawa-Tachino R, Kabuyama N, Gotoh T, Kagei S, Naruse M, Kisu Y, Togashi T, Sugano S, Usami H, Nomura N. 2003. High-throughput classification of images of cells transfected with cDNA clones. *C R Biol* **326:** (10-11) 993.

Mobasheri A, Airley R, Foster CS, Schulze-Tanzil G, Shakibaei M. 2004. Post-genomic applications of tissue microarrays: Basic research, prognostic oncology, clinical genomics and drug discovery. *Histol Histopathol* **19:** (1) 325.

Mocellin S, Rossi CR, Traldi P, Nitti D, Lise M. 2004. Molecular oncology in the post-genomic era: The challenge of proteomics (Review). *Trends Mol Med* **10:** (1) 24.

Nagase T, Kikuno R, Ohara O. 2003. The Kazusa cDNA project for identification of unknown human transcripts (Review). *C R Biol* **326:** (10-11) 959.

Ruan YJ, Le Ber P, Ng HH, Liu ET. 2004. Interrogating the transcriptome (Review). *Trends Biotechnol* **22:** (1) 23.

Sasaki T. 2003. Rice genome analysis: Understanding the genomic secrets of the rice plant (Review). *Breeding Sci* **53:** (4) 281.

Shatkay H, Feldman R. 2003. Mining the biomedical literature in the genomic era: An overview. *J Comput Biol* **10:** (6) 821.

Simon R, Mirlacher M, Sauter G. 2004. Tissue microarrays (Review). *Biotechniques* **36:** (1) 98.

Simon R. 2003. Using DNA microarrays for diagnostic and prognostic prediction. *Expert Rev Mol Diagn* **3:** (5) 587.

Smith CV, Sacchettini JC. 2003. *Mycobacterium tuberculosis*: A model system for structural genomics. *Curr Opin Struct Biol* **13:** (6) 658.

Sparre T, Bergholdt R, Nerup J, Pociot F. 2003. Application of genomics and proteomics in type 1 diabetes pathogenesis research. *Expert Rev Mol Diagn* **3:** (6) 743.

Turk BE, Cantley LC. 2004. Peptide libraries: At the crossroads of proteomics and bioinformatics. *Drug Discov Today* **9:** (Suppl 2) S47.

Weinstein JN, Pommier Y. 2003. Transcriptomic analysis of the NCI-60 cancer cell lines (Review). *C R Biol* **326:** (10-11) 909.

Wiseman A. 2004. Novel food-proteomics by control of bioprocess-enzymology outcomes (Review). *Trends Food Sci Technol* **15:** (1) 44.

Zhang HG, Xiu RJ. 2003. Micro-vascular medicine and proteomics. *Clin Hemorheol Microcirc* **29:** (3-4) 189.

### 3 Large-scale sequencing and mapping

Larimer FW, Chain P, Hauser L, Lamerdin J, Malfatti S, Do L, Land ML, Pelletier DA, Beatty JT, Lang AS *et al*. 2004. Complete genome sequence of the metabolically versatile photosynthetic bacterium *Rhodopseudomonas palustris*. *Nat Biotechnol* **22:** (1) 55.

Ota T, Suzuki Y, Nishikawa T, Otsuki T, Sugiyama T, Irie R, Wakamatsu A, Hayashi K, Sato H, Nagai K *et al*. 2004. Complete sequencing and characterization of 21,243 full-length human cDNAs. *Nat Genet* **36:** (1) 40.

Sesterhenn TM, Slaven BE, Smulian AG, Cushion MT. 2003. Generation of sequencing libraries for the *Pneumocystis* genome project. *J Eukaryot Microbiol* **50:** (S) 663.

Wang PW, Chu L, Guttman DS. 2004. Complete sequence and evolutionary genomic analysis of the *Pseudomonas aeruginosa* transposable bacteriophage D3112. *J Bacteriol* **186:** (2) 400.

Zimdahl H, Nyakatura G, Brandt P, Schulz H, Hummel O, Fartmann B, Brett D, Droege M, Monti J, Lee YA *et al*. 2004. A SNP map of the rat genome generated from cDNA sequences. *Science* **303:** (5659) 807.

### 4 Evolutionary genomics

Amores A, Suzuki T, Yan YL, Pomeroy J, Singer A, Amemiya C, Postlethwait JH. 2004. Developmental roles of pufferfish *Hox* clusters and genome evolution in ray-fin fish. *Genome Res* **14:** (1) 1.

Chiu CH, Dewar K, Wagner GP, Takahashi K, Ruddle F, Ledje C, Bartsch P, Scemama JL, Stellwag E, Fried C *et al*. 2004. Bichir *Hox*A cluster sequence reveals surprising trends in ray-finned fish genomic evolution. *Genome Res* **14:** (1) 11.

Doumith M, Cazalet C, Simoes N, Frangeul L, Jacquet C, Kunst F, Martin P, Cossart P, Glaser P, Buchrieser C. 2004. New aspects regarding evolution and virulence of *Listeria monocytogenes* revealed by comparative genomics and DNA arrays. *Infect Immun* **72:** (2) 1072.

Oshima K, Kakizawa S, Nishigawa H, Jung HY, Wei W, Suzuki S, Arashida R, Nakata D, Miyata S, Ugaki M *et al*. 2004. Reductive evolution suggested from the complete genome sequence of a plant-pathogenic phytoplasma. *Nat Genet* **36:** (1) 27.

Qi J, Wang B, Hao BI. 2004. Whole proteome prokaryote phylogeny without sequence alignment: A K-string composition approach. *J Mol Evol* **58:** (1) 1.

Von Mering C, Zdobnov EM, Tsoka S, Ciccarelli FD, Pereira-Leal JB, Ouzounis CA, Bork P. 2003. Genome evolution reveals biochemical networks and functional modules. *Proc Natl Acad Sci U S A* **100:** (26) 15428.

Wolf YI, Rogozin IB, Koonin EV. 2004. Coelomata and not ecdysozoa: Evidence from genome-wide phylogenetic analysis. *Genome Res* **14:** (1) 29.

### 5 Comparative genomics

Ferreira A, Gray M, Wiedmann M, Boor KJ. 2004. Comparative genomic analysis of the sigB operon in *Listeria monocytogenes* and in other Gram-positive bacteria. *Curr Microbiol* **48:** (1) 39.

Ge H, Chuang YYE, Zhao SP, Tong M, Tsai MH, Temenak JJ, Richards AL, Ching WM. 2004. Comparative genomics of *Rickettsia prowazekii* Madrid E and Breinl strains. *J Bacteriol* **186:** (2) 556.

Islas S, Becerra A, Luisi PL, Lazcano A. 2004. Comparative genomics and the gene complement of a minimal cell. *Orig Life Evol Biosph* **34:** (1-2) 243.

McCarroll SA, Murphy CT, Zou SG, Pletcher SD, Chin CS, Jan YN, Kenyon C, Bargmann CI, Li H. 2004. Comparing genomic expression patterns across species identifies shared transcriptional profile in aging. *Nat Genet* **36:** (2) 197.

Nobrega MA, Pennacchio LA. 2004. Comparative genomic analysis as a tool for biological discovery. *J Physiol* **554:** (1) 31.

Wiedenheft B, Stedman K, Roberto F, Willits D, Gleske AK, Zoeller L, Snyder J, Douglas T, Young M. 2004. Comparative genomic analysis of hyperthermophilic archaeal Fusellovitidae viruses. *J Virol* **78:** (4) 1954.

Zhang R, Zhang CT. 2003. Identification of genomic islands in the genome of *Bacillus cereus* by comparative analysis with *Bacillus anthracis*. *Physiol Genomics* **16:** (1) 19.

### 6 Pathways, gene families and regulons

Baud S, Vaultier MN, Rochat C. 2004. Structure and expression profile of the sucrose synthase multigene family in *Arabidopsis*. *J Exp Bot* **55:** (396) 397.

Copyright © 2004 John Wiley & Sons, Ltd.

Kofuji R, Sumikawa N, Yamasaki M, Kondo K, Ueda K, Ito M, Hasebe M. 2003. Evolution and divergence of the MADS-box gene family based on genome-wide expression analyses. *Mol Biol Evol* **20:** (12) 1963.

Schuller C, Mamnun YM, Mollapour M, Krapf G, Schuster M, Bauer BE, Piper PW, Kuchler K. 2004. Global phenotypic analysis and transcriptional profiling defines the weak acid stress response regulon in S*accharomyces cerevisiae*. *Mol Biol Cell* **15:** (2) 706.

### 7 Pharmacogenomics

Agrawal D, Chen T, Irby R, Quackenbush J, Chambers AF, Szabo M, Cantor A, Coppola D, Yeatman TJ. 2003. Osteopontin identified as colon cancer tumor progression marker. *C R Biol* **326:** (10-11) 1041.

Ahn WS, Kim KW, Bae SM, Yoon JH, Lee JM, Namkoong SE, Kim JH, Kim CK, Lee YJ, Kim YW. 2003. Targeted cellular process profiling approach for uterine leiomyoma using cDNA microarray, proteomics and gene ontology analysis. *Int J Exp Pathol* **84:** (6) 267.

Bruchova H, Kalinova M, Brdicka R. 2004. Array-based analysis of gene expression in childhood acute lymphoblastic leukemia. *Leuk Res* **28:** (1) 1.

Bueno R, Loughlin KR, Powell MH, Gordon GJ. 2004. A diagnostic test for prostate cancer from gene expression profiling data. *J Urol* **171:** (2) 903.

Cecconi D, Astner H, Donadelli M, Palmieri M, Missiaglia E, Hamdan M, Scarpa A, Righetti PG. 2003. Proteomic analysis of pancreatic ductal carcinoma cells treated with 5-aza-2′-deoxycytidine. *Electrophoresis* **24:** (24) 4291.

Chen HW, Yu SL, Chen JJW, Li HN, Lin YC, Yao PL, Chou HY, Chien CT, Chen WJ, Lee YT *et al*. 2004. Anti-invasive gene expression profile of curcumin in lung adenocarcinoma based on a high throughput microarray analysis. *Mol Pharmacol* **65:** (1) 99.

Cho WCS, Yip TTC, Yip C, Yip V, Thulasiraman V, Ngan RKC, Yip TT, Lau WH, An JSK, Law SCK *et al*. 2004. Identification of serum amyloid a protein as a potentially useful biomarker to monitor relapse of nasopharyngeal cancer by serum proteomic profiling. *Clin Cancer Res* **10:** (1) 43.

Daibata M, Matsuo Y, Machida H, Taguchi T, Ohtsuki Y, Taguchi H. 2004. Differential gene-expression profiling in the leukemia cell lines derived from indolent and aggressive phases of $CD56^+$ T-cell large granular lymphocyte leukemia. *Int J Cancer* **108:** (6) 845.

Dick DM, Li TK, Edenberg HJ, Hesselbrock V, Kramer J, Kuperman S, Porjesz B, Bucholz K, Goate A, Nurnberger J *et al*. 2004. A genome-wide screen for genes influencing conduct disorder. *Mol Psychiatry* **9:** (1) 81.

Dooley TP, Curto EV, Reddy SP, Davis RL, Lambert GW, Wilborn TW, Elson CO. 2004. Regulation of gene expression in inflammatory bowel disease and correlation with IBD drugs - Screening by DNA microarrays. *Inflamm Bowel Dis* **10:** (1) 1.

Fargiano AA, Desai KV, Green JE. 2003. Interrogating mouse mammary cancer models: Insights from gene expression profiling. *J Mammary Gland Biol Neoplasia* **8:** (3) 321.

Godl K, Wissing J, Kurtenbach A, Habenberger P, Blencke S, Gutbrod H, Salassidis K, Stein-Gerlach M, Missio A, Cotten M *et al*. 2003. An efficient proteomics method to identify the cellular targets of protein kinase inhibitors. *Proc Natl Acad Sci U S A* **100:** (26) 15434.

Green JE, Desai K, Ye YM, Kavanaugh C, Calvo A, Huh JI. 2004. Genomic approaches to understanding mammary tumor progression in transgenic mice and responses to therapy. *Clin Cancer Res* **10:** (1 Pt 2) 385S.

Greshock J, Naylor TL, Margolin A, Diskin S, Cleaver SH, Futreal PA, de Jong PJ, Zhao SY, Liebman M, Weber BL. 2004. 1-Mb resolution array-based comparative genomic hybridization using a BAC clone set optimized for cancer gene analysis. *Genome Res* **14:** (1) 179.

He QY, Chen J, Kung HF, Yuen APW, Chiu JF. 2004. Identification of tumor-associated proteins in oral tongue squamous cell carcinoma by proteomics. *Proteomics* **4:** (1) 271.

Iwata SI, Nomoto M, Morioka H, Miyata A. 2004. Gene expression profiling in the midbrain of striatal 6-hydroxydopamine-injected mice. *Synapse* **51:** (4) 279.

Joo WA, Kang MJ, Son WK, Lee HJ, Lee DY, Lee E, Kim CW. 2003. Monitoring protein expression by proteomics: Human plasma exposed to benzene. *Proteomics* **3:** (12) 2402.

Kang HC, Kim IJ, Park JH, Shin Y, Ku JL, Jung MS, Yoo BC, Kim HK, Park JG. 2004. Identification of genes with differential expression in acquired drug-resistant gastric cancer cells using high-density oligonucleotide microarrays. *Clin Cancer Res* **10:** (1) 272.

Khawaja X, Xu J, Liang JJ, Barrett JE. 2004. Proteomic analysis of protein changes developing in rat hippocampus after chronic antidepressant treatment: Implications for depressive disorders and future therapies. *J Neurosci Res* **75:** (4) 451.

Kim CH, Kim DK, Choi SJ, Choi KH, Song KS, Chi J, Ko JS, Hwang SY, Seong JK. 2003. Proteomic and transcriptomic analysis of interleukin-1β treated lung carcinoma cell line. *Proteomics* **3:** (12) 2454.

Lapointe J, Li C, Higgins JP, Van de Rijn M, Bair E, Montgomery K, Ferrari M, Egevad L, Rayford W, Begerheim U *et al*. 2004. Gene expression profiling identifies clinically relevant subtypes of prostate cancer. *Proc Natl Acad Sci U S A* **101:** (3) 811.

Lee CL, Hsiao HH, Lin CW, Wu SP, Huang SY, Wu CY, Wang AHJ, Khoo KW. 2003. Strategic shotgun proteomics approach for efficient construction of an expression map of targeted protein families in hepatoma cell lines. *Proteomics* **3:** (12) 2472.

Leonard P, Sharp T, Henderson S, Hewitt D, Pringle J, Sandison A, Goodship A, Whelan J, Boshoff C. 2003. Gene expression array profile of human osteosarcoma. *Br J Cancer* **89:** (12) 2284.

Li AL, Li HW, Jin G, Xiu RJ. 2003. A proteomic study on cell cycle progression of endothelium exposed to tumor conditioned medium and the possible role of cyclin D-1/E. *Clin Hemorheol Microcirc* **29:** (3-4) 383.

Li MH, Lin YM, Hasegawa S, Shimokawa T, Murata K, Kameyama M, Ishikawa O, Katagiri T, Tsunoda T, Nakamura Y *et al*. 2004. Genes associated with liver metastasis of colon cancer, identified by genome-wide cDNA microarray. *Int J Oncol* **24:** (2) 305.

Li QS, Schacker T, Carlis J, Beilman G, Nguyen P, Haase AT. 2004. Functional genomic analysis of the response of HIV-1-infected lymphatic tissue to antiretroviral therapy. *J Infect Dis* **189:** (4) 572.

Lilien RH, Farid H, Donald BR. 2003. Probabilistic disease classification of expression-dependent proteomic data from mass spectrometry of human serum. *J Comput Biol* **10:** (6) 925.

Liu J, Dehbi M, Moeck G, Arhin F, Bauda P, Bergeron D, Callejo M, Ferretti V, Ha NH, Kwan T *et al*. 2004. Antimicrobial drug discovery through bacteriophage genomics. *Nat Biotechnol* **22:** (2) 185.

Loni L, De Braud F, Zinzani PL, Danesi R. 2003. Pharmacogenetics and proteomics of anticancer drugs in non-Hodgkin's lymphoma. *Leuk Lymphoma* **44:** (Suppl 3) S115.

Lum PY, Armour CD, Stepaniants SB, Cavet G, Wolf MK, Butler JS, Hinshaw JC, Garnier P, Prestwich GD, Leonardson A *et al*. 2004. Discovering modes of action for therapeutic compounds using a genome-wide screen of yeast heterozygotes. *Cell* **116:** (1) 121.

Maitra A, Hansel DE, Argani P, Ashfaq R, Rahman A, Naji A, Deng SP, Geradts J, Hawthorne L, House MG *et al*. 2003. Global expression analysis of well-differentiated pancreatic endocrine neoplasms using oligonucleotide microarrays. *Clin Cancer Res* **9:** (16) 5988.

Martin DB, Gifford DR, Wright ME, Keller A, Yi E, Goodlett DR, Aebersold R, Nelson PS. 2004. Quantitative proteomic analysis of proteins released by neoplastic prostate epithelium. *Cancer Res* **64:** (1) 347.

Miller I, Teinfalt M, Leschnik M, Wait R, Gemeiner M. 2004. Nonreducing two-dimensional gel electrophoresis for the detection of Bence Jones proteins in serum and urine. *Proteomics* **4:** (1) 257.

Park M, Kang H, Lim S, Lee CT, Kim O. 2003. Differential protein expressions induced by adenovirus-mediated p16 gene transfer into Balb/c nude mouse. *Proteomics* **3:** (12) 2412.

Poland J, Urbani A, Lage H, Schnolzer M, Sinha P. 2004. Study of the development of thermoresistance in human pancreatic carcinoma cell lines using proteome analysis. *Electrophoresis* **25:** (1) 173.

Price N. 2003. Gene expression profiling as a diagnostic and prognostic tool in B-cell non-Hodgkin's lymphoma. *Clin Lymphoma* **4:** (3) 149.

Rautajoki K, Nyman TA, Lahesmaa R. 2004. Proteome characterization of human T helper 1 and 2 cells. *Proteomics* **4:** (1) 84.

Raza SM, Fuller GN, Rhee CH, Huang SY, Hess K, Zhang W, Sawaya R. 2004. Identification of necrosis-associated genes in glioblastoma by cDNA microarray analysis. *Clin Cancer Res* **10:** (1) 212.

Rosenwald A, Staudt LM. 2003. Gene expression profiling of diffuse large B-cell lymphoma. *Leuk Lymphoma* **44:** (Suppl 3) S41.

Schaub S, Rush D, Wilkins J, Gibson IW, Weiler T, Sangster K, Nicolle

Copyright © 2004 John Wiley & Sons, Ltd. 

L, Karpinski M, Jeffry J, Nickerson P. 2004. Proteomic based detection of urine proteins associated with acute renal allograft rejection. *J Am Soc Nephrol* **15:** (1) 219.
Schwaenen C, Nessling M, Wessendorf S, Salvi T, Wrobel G, Radlwimmer B, Kestler HA, Haslinger C, Stilgenbauer S, Dohner H *et al*. 2004. Automated array-based genomic profiling in chronic lymphocytic leukemia: Development of a clinical tool and discovery of recurrent genomic alterations. *Proc Natl Acad Sci U S A* **101:** (4) 1039.
Son WK, Lee DY, Lee SH, Joo WA, Kim CW. 2003. Analysis of proteins expressed in rat plasma exposed to dioxin using 2-dimensional gel electrophoresis. *Proteomics* **3:** (12) 2393.
Sprenger RR, Speijer D, Back JW, De Koster CG, Pannekoek H, Horrevoets AJG. 2004. Comparative proteomics of human endothelial cell caveolae and rafts using two-dimensional gel electrophoresis and mass spectrometry. *Electrophoresis* **25:** (1) 156.
Stevens EV, Liotta LA, Kohn EC. 2003. Proteomic analysis for early detection of ovarian cancer: A realistic approach? *Int J Gynecol Cancer* **13:** (Suppl 2) 133.
Strominger JL, Byrne MC, Wilson SB. 2003. Regulation of dendritic cell subsets by NKT cells. *C R Biol* **326:** (10-11) 1045.
Swamy SMK, Tan P, Zhu YZ, Lu J, Achuth HN, Moochhala S. 2004. Role of phenytoin in wound healing: Microarray analysis of early transcriptional responses in human dermal fibroblasts. *Biochem Biophys Res Commun* **314:** (3) 661.
Takashima M, Kuramitsu Y, Yokoyama Y, Iizuka N, Toda T, Sakaida I, Okita K, Oka M, Nakamura K. 2003. Proteomic profiling of heat shock protein 70 family members as biomarkers for hepatitis C virus-related hepatocellular carcinoma. *Proteomics* **3:** (12) 2487.
Towbin H, Bair KW, DeCaprio JA, Eck MJ, Kim S, Kinder FR, Morollo A, Mueller DR, Schindler P, Song HK *et al*. 2003. Proteomics-based target identification: Bengamides as a new class of methionine aminopeptidase inhibitors. *J Biol Chem* **278:** (52) 52964.
Tsubakihara M, Williams NK, Keogh A, Dos Remedios CG. 2004. Comparison of gene expression between left atria and left ventricles from non-diseased humans. *Proteomics* **4:** (1) 261.
Turck N, Richert S, Gendry P, Stutzmann J, Kedinger M, Leize E, Simon-Assmann P, Van Dorsselaer A, Launay JF. 2004. Proteomic analysis of nuclear proteins from proliferative and differentiated human colonic intestinal epithelial cells. *Proteomics* **4:** (1) 93.
Unami A, Shinohara Y, Kajimoto K, Baba Y. 2004. Comparison of gene expression profiles between white and brown adipose tissues of rat by microarray analysis. *Biochem Pharmacol* **67:** (3) 555.
Van Balkom BWM, Hoffert JD, Chou CL, Knepper MA. 2004. Proteomics analysis of long-term vasopressin action in the inner medullary collecting duct of the Brattleboro rat. *Am J Physiol* **286:** (2) F215.
Weigelt B, Glas AM, Wessels LFA, Witteveen AT, Peterse JL, Van't Veert LJ. 2003. Gene expression profiles of primary breast tumors maintained in distant metastases. *Proc Natl Acad Sci U S A* **100:** (26) 15901.
Wheelock AM, Zhang L, Tran MU, Morin D, Penn S, Buckpitt AR, Plopper CG. 2004. Isolation of rodent airway epithelial cell proteins facilitates *in vivo* proteomics studies of lung toxicity. *Am J Physiol* **286:** (2) L399.
Whiteside MA, Chen DT, Desmond RA, Abdulkadir SA, Johanning GL. 2004. A novel time-course cDNA microarray analysis method identifies genes associated with the development of cisplatin resistance. *Oncogene* **23:** (3) 744.
Won Y, Song HJ, Kang TW, Kim JJ, Han BD, Lee SW. 2003. Pattern analysis of serum proteome distinguishes renal cell carcinoma from other urologic diseases and healthy persons. *Proteomics* **3:** (12) 2310.
Xiao Z, Conrads TP, Lucas DA, Janini GM, Schaefer CF, Buetow KH, Issaq HJ, Veenstra TD. 2004. Direct ampholyte-free liquid-phase isoelectric peptide focusing: Application to the human serum proteome. *Electrophoresis* **25:** (1) 128.
Yu JH, Yun SY, Lim JW, Kim H, Kim KH. 2003. Proteome analysis of rat pancreatic acinar cells: Implications for cerulein-induced acute pancreatitis. *Proteomics* **3:** (12) 2446.
Zhang RL, Barker L, Pinchev D, Marshall J, Rasamoelisolo M, Smith C, Kupchak P, Kireeva I, Ingratta L, Jackowski G. 2004. Mining biomarkers in human sera using proteomic tools. *Proteomics* **4:** (1) 244.

### 8 EST, cDNA and other clone resources

Connor EE, Sonstegard TS, Keele JW, Bennett GL, Williams JL, Papworth R, Van Tassell CP, Ashwell MS. 2004. Physical and linkage mapping of mammary-derived expressed sequence tags in cattle. *Genomics* **83:** (1) 148.
Kuhl JC, Cheung F, Yuan QP, Martin W, Zewdie Y, McCallum J, Catanach A, Rutherford P, Sink KC, Jenderek M *et al*. 2004. A unique set of 11,008 onion expressed sequence tags reveals expressed sequence and genomic differences between the monocot orders Asparagales and Poales. *Plant Cell* **16:** (1) 114.
Kwon KH, Kim M, Kim JY, Kim KW, Kim SI, Park YM, Yoo JS. 2003. Efficiency improvement of peptide identification for an organism without complete genome sequence, using expressed sequence tag database and tandem mass spectral data. *Proteomics* **3:** (12) 2305.
Nam MH, Heo EJ, Kim JY, Kim SI, Kwon KH, Seo JB, Kwon O, Jong SY, Park YM. 2003. Proteome analysis of the responses of *Panax ginseng* C.A. Meyer leaves to high light: Use of electrospray ionization quadrupole-time of flight mass spectrometry and expressed sequence tag data. *Proteomics* **3:** (12) 2351.
Nobis W, Ren XN, Suchyta SP, Suchyta TR, Zanella AJ, Coussens PM. 2003. Development of a porcine brain cDNA library, EST database, and microarray resource. *Physiol Genomics* **16:** (1) 153.
Sims AH, Robson GD, Hoyle DC, Oliver SG, Turner G, Prade RA, Russell HH, Dunn-Coleman NS, Gent ME. 2004. Use of expressed sequence tag analysis and cDNA microarrays of the filamentous fungus *Aspergillus nidulans*. *Fungal Genet Biol* **41:** (2) 199.
Sturzenbaum SR, Parkinson J, Blaxter M, Morgan AJ, Kille P, Georgiev O. 2003. The earthworm expressed sequence tag project. *Pedobiologia* **47:** (5-6) 447.
Yamashita R, Suzuki Y, Nakai K, Sugano S. 2003. Small open reading frames in 5′ untranslated regions of mRNAs. *C R Biol* **326:** (10-11) 987.

### 9 Functional genomics

Biden TJ, Robinson D, Cordery D, Hughes WE, Busch AK. 2004. Chronic effects of fatty acids on pancreatic β-cell function - New insights from functional genomics. *Diabetes* **53:** (Suppl 1) S159.
Boutros M, Kiger AA, Armknecht S, Kerr K, Hild M, Koch B, Haas SA, Paro R, Perrimon N. 2004. Genome-wide RNAi analysis of growth and viability in *Drosophila* cells. *Science* **303:** (5659) 832.
Devlin PF, Yanovsky MJ, Kay SA. 2003. A genomic analysis of the shade avoidance response in *Arabidopsis*. *Plant Physiol* **133:** (4) 1617.
Eisenhaber B, Wildpaner M, Schultz CJ, Borner GHH, Dupree P, Eisenhaber F. 2003. Glycosylphosphatidylinositol lipid anchoring of plant proteins. Sensitive prediction from sequence- and genome-wide studies for *Arabidopsis* and rice. *Plant Physiol* **133:** (4) 1691.
Gan L, Ye SM, Chu A, Anton K, Yi SL, Vincent VA, Von Schack D, Chin D, Murray J, Lohr S *et al*. 2004. Identification of cathepsin B as a mediator of neuronal death induced by Aβ-activated microglial cells using a functional genomics approach. *J Biol Chem* **279:** (7) 5565.
Giaever G, Flaherty P, Kumm J, Proctor M, Nislow C, Jaramillo DF, Chu AM, Jordan MI, Arkin AP, Davis RW. 2004. Chemogenomic profiling: Identifying the functional interactions of small molecules in yeast. *Proc Natl Acad Sci U S A* **101:** (3) 793.
Hanke GT, Kimata-Ariga Y, Taniguchi I, Hase T. 2004. A post genomic characterization of *Arabidopsis* ferredoxins. *Plant Physiol* **134:** (1) 255.
Jim K, Parmar K, Singh M, Tavazoie S. 2004. A cross-genomic approach for systematic mapping of phenotypic traits to genes. *Genome Res* **14:** (1) 109.
Lee S, Kim J, Son JS, Nam J, Jeong DH, Lee K, Jang S, Yoo J, Lee J, Lee DY *et al*. 2003. Systematic reverse genetic screening of T-DNA tagged genes in rice for functional genomic analyses: MADS-box genes as a test case. *Plant Cell Physiol* **44:** (12) 1403.
McBride MW, Charchar FJ, Graham D, Miller WH, Strahorn P, Carr FJ, Dominiczak AF. 2004. Functional genomics in rodent models of hypertension. *J Physiol* **554:** (1) 56.
Seta KA, Millhorn DE. 2004. Functional genomics approach to hypoxia signaling. *J Appl Physiol* **96:** (2) 765.
Stuitje AR, Verbree EC, Van der Linden KH, Mietkiewska EM, Nap JP,

Copyright © 2004 John Wiley & Sons, Ltd. 

Kneppers TJA. 2003. Seed-expressed fluorescent proteins as versatile tools for easy (co)transformation and high-throughput functional genomics in *Arabidopsis*. *Plant Biotechnol J* **1:** (4) 301.
Tong AHY, Lesage G, Bader GD, Ding HM, Xu H, Xin XF, Young J, Berriz GF, Brost RL, Chang M *et al*. 2004. Global mapping of the yeast genetic interaction network. *Science* **303:** (5659) 808.
Wei HC, Shu HD, Price JV. 2003. Functional genomic analysis of the 61D-61F region of the third chromosome of *Drosophila melanogaster*. *Genome* **46:** (6) 1049.

### I0 Transcriptomics

Belland RJ, Nelson DE, Virok D, Crane DD, Hogan D, Sturdevant D, Beatty WL, Caldwell HD. 2003. Transcriptome analysis of chlamydial growth during IFN-γ-mediated persistence and reactivation. *Proc Natl Acad Sci U S A* **100:** (26) 15971.
Bortoli S, Rehault V, Eveno E, Auffray C, Butler-Browne G, Pietu G. 2003. Gene expression profiling of human satellite cells during muscular aging using cDNA arrays. *Gene* **321:** 145.
Calvo E, Andersen J, Francischetti IM, Del Capurro M, DeBianchi AG, James AA, Ribeiro JMC, Marinotti O. 2004. The transcriptome of adult female *Anopheles darlingi* salivary glands. *Insect Mol Biol* **13:** (1) 73.
Di Pietro M, Marra G, Cejka P, Stojic L, Menigatti M, Cattaruzza MS, Jiricny J. 2003. Mismatch repair-dependent transcriptome changes in human cells treated with the methylating agent *N*-methyl-*N′*-nitro-*N*-nitrosoguanidine. *Cancer Res* **63:** (23) 8153.
Fizames C, Munos S, Cazettes C, Nacry P, Boucherez J, Gaymard F, Piquemal D, Delorme V, Commes TS, Doumas P *et al*. 2004. The *Arabidopsis* root transcriptome by serial analysis of gene expression. Gene identification using the genome sequence. *Plant Physiol* **134:** (1) 67.
Gibbings JG, Cook BP, Dufault MR, Madden SL, Khuri S, Turnbull CJ, Dunwell JM. 2003. Global transcript analysis of rice leaf and seed using SAGE technology. *Plant Biotechnol J* **1:** (4) 271.
Herath CB, Shiojima S, Ishiwata H, Katsuma S, Kadowaki T, Ushizawa K, Imai K, Takahashi T, Hirasawa A, Tsujimoto G *et al*. 2004. Pregnancy-associated changes in genome-wide gene expression profiles in the liver of cow throughout pregnancy. *Biochem Biophys Res Commun* **313:** (3) 666.
Higgins VJ, Rogers PJ, Dawes IW. 2003. Application of genome-wide expression analysis to identify molecular markers useful in monitoring industrial fermentations. *Appl Environ Microbiol* **69:** (12) 7535.
Hudson ME, Quail PH. 2003. Identification of promoter motifs involved in the network of phytochrome A-regulated gene expression by combined analysis of genomic sequence and microarray data. *Plant Physiol* **133:** (4) 1605.
Jenson SD, Robetorye RS, Bohling SD, Schumacher JA, Morgan JW, Lim MS, Elenitoba-Johnson KSJ. 2003. Validation of cDNA microarray gene expression data obtained from linearly amplifield RNA. *J Clin Pathol Mol Pathol* **56:** (6) 307.
Jiao YL, Yang HJ, Ma LG, Sun N, Yu HY, Liu T, Gao Y, Gu HY, Chen ZL, Wada M *et al*. 2003. A genome-wide analysis of blue-light regulation of *Arabidopsis* transcription factor gene expression during seedling development. *Plant Physiol* **133:** (4) 1480.
Junghans P, Kaehne T, Beyer M, Metges CC, Schwerin M. 2004. Dietary protein-related changes in hepatic transcription correspond to modifications in hepatic protein expression in growing pigs. *J Nutr* **134:** (1) 43.
Kao KC, Yang YL, Boscolo R, Sabatti C, Roychowdhury V, Liao JC. 2004. Transcriptome-based determination of multiple transcription regulator activities in *Escherichia coli* by using network component analysis. *Proc Natl Acad Sci U S A* **101:** (2) 641.
Kim HJ, Ishidou E, Kitagawa E, Momose Y, Iwahashi H. 2004. A yeast DNA microarray for the evaluation of toxicity in environmental water containing burned ash. *Environ Monit Assess* **92:** (1-3) 253.
Kim SK, Wang KC, Hong SJ, Chung CK, Lim SY, Kim YY, Chi JG, Kim CJ, Chung YN, Kim HJ *et al*. 2003. Gene expression profile analyses of cortical dysplasia by cDNA arrays. *Epilepsy Res* **56:** (2-3) 175.
Kreps J, Budworth P, Goff S, Wang RL. 2003. Identification of putative plant cold responsive regulatory elements by gene expression profiling and a pattern enumeration algorithm. *Plant Biotechnol J* **1:** (5) 345.
Li Y, Li T, Liu SZ, Qiu MY, Han ZY, Jiang ZL, Li RY, Ying K, Xie Y, Mao YM. 2004. Systematic comparison of the fidelity of aRNA, mRNA and T-RNA on gene expression profiling using cDNA microarray. *J Biotechnol* **107:** (1) 19.
Narasimhan S, Camaino MJ, Liang FT, Santiago F, Laskowski M, Philipp MT, Pachner AR, Radolf JD, Fikrig E. 2003. *Borrelia burgdorferi* transcriptome in the central nervous system of non-human primates. *Proc Natl Acad Sci U S A* **100:** (26) 15953.
O'Rourke SM, Herskowitz I. 2004. Unique and redundant roles for HOG MAPK pathway components as revealed by whole-genome expression analysis. *Mol Biol Cell* **15:** (2) 532.
Palma M, DeLuca D, Worgall S, Quadri LEN. 2004. Transcriptome analysis of the response of *Pseudomonas aeruginosa* to hydrogen peroxide. *J Bacteriol* **186:** (1) 248.
Richards M, Tan SP, Tan JH, Chan WK, Bongso A. 2003. The transcriptome profile of human embryonic stem cells as defined by SAGE. *Stem Cells* **22:** (1) 51.
Sakabe NJ, De Souza JES, Galante PAF, De Oliveira PSL, Passetti F, Brentani H, Osorio EC, Zaiats AC, Leerkes MR, Kitajima JP *et al*. 2003. ORESTES are enriched in rare exon usage variants affecting the encoded proteins. *C R Biol* **326:** (10-11) 979.
Schmitt WA, Stephanopoulos G. 2003. Prediction of transcriptional profiles of *Synechocystis* PCC6803 by dynamic autoregressive modeling of DNA microarray data. *Biotechnol Bioeng* **84:** (7) 855.
Suchyta SP, Sipkovsky S, Halgren RG, Kruska R, Elftman M, Weber-Nielsen M, Vandehaar MJ, Xiao L, Tempelman RJ, Coussens PM. 2003. Bovine mammary gene expression profiling using a cDNA microarray enhanced for mammary-specific transcripts. *Physiol Genomics* **16:** (1) 8.
Tsoi SCM, Cale JM, Bird IM, Ewart V, Brown LL, Douglas S. 2003. Use of human cDNA microarrays for identification of differentially expressed genes in Atlantic salmon liver during *Aeromonas salmonicida* infection. *Mar Biotechnol* **5:** (6) 545.
Watson A, Mata J, Bahler J, Carr A, Humphrey T. 2004. Global gene expression responses of fission yeast to ionizing radiation. *Mol Biol Cell* **15:** (2) 851.

### II Proteomics

Baggerman G, Clynen E, Huybrechts J, Verleyen P, Clerens S, De Loof A, Schoofs L. 2003. Peptide profiling of a single *Locusta migratoria* corpus cardiacum by nano-LC tandem mass spectrometry. *Peptides* **24:** (10) 1475.
Baudouin-Cornu P, Schuerer K, Marliere P, Thomas D. 2004. Intimate evolution of proteins - Proteome atomic content correlates with genome base composition. *J Biol Chem* **279:** (7) 5421.
Blonder J, Conrads TP, Yu LR, Terunuma A, Janini GM, Issaq HJ, Vogel JC, Veenstra TD. 2004. A detergent- and cyanogen bromide-free method for integral membrane proteomics: Application to *Halobacterium* purple membranes and the human epidermal membrane proteome. *Proteomics* **4:** (1) 31.
Bunai K, Ariga M, Inoue T, Nozaki M, Ogane S, Kakeshita H, Nemoto T, Nakanishi H, Yamane K. 2004. Profiling and comprehensive expression analysis of ABC transporter solute-binding proteins of *Bacillus subtilis* membrane based on a proteomic approach. *Electrophoresis* **25:** (1) 141.
Chan LL, Hodgkiss IJ, Lu S, Lo SCL. 2004. Use of two-dimensional gel electrophoresis proteome reference maps of dinoflagellates for species recognition of causative agents of harmful algal blooms. *Proteomics* **4:** (1) 180.
Cho CW, Lee SH, Choi J, Park SJ, Ha DJ, Kim HJ, Kim CW. 2003. Improvement of the two-dimensional gel electrophoresis analysis for the proteome study of *Halobacterium salinarum*. *Proteomics* **3:** (12) 2325.
Corpillo D, Gardini G, Vaira AM, Basso M, Aime S, Accotto GR, Fasano M. 2004. Proteomics as a tool to improve investigation of substantial equivalence in genetically modified organisms: The case of a virus-resistant tomato. *Proteomics* **4:** (1) 193.
Dickinson DN, La Duc MT, Haskins WE, Gornushkin I, Winefordner D, Powell DH, Venkateswaran K. 2004. Species differentiation of a diverse suite of *Bacillus* spores by mass spectrometry-based protein profiling. *Appl Environ Microbiol* **70:** (1) 475.
Friso G, Giacomelli L, Ytterberg AJ, Peltier JB, Rudella A, Sun Q, van Wijk KJ. 2004. In-depth analysis of the thylakoid membrane proteome of *Arabidopsis thaliana* chloroplasts: New proteins, new functions, and

Copyright © 2004 John Wiley & Sons, Ltd. 

a plastid proteome database. *Plant Cell* **16:** (2) 478.
Heazlewood JL, Tonti-Filippini JS, Gout AM, Day DA, Whelan J, Millar AH. 2004. Experimental analysis of the *Arabidopsis* mitochondrial proteome highlights signaling and regulatory components, provides assessment of targeting prediction programs, and indicates plant-specific mitochondrial proteins. *Plant Cell* **16:** (1) 241.
Herranen M, Battchikova N, Zhang PP, Graf A, Sirpio S, Paakkarinen V, Aro EM. 2004. Towards functional proteomics of membrane protein complexes in S*ynechocystis* sp PCC 6803. *Plant Physiol* **134:** (1) 470.
Kikuchi M, Hatano N, Yokota S, Shimozawa N, Imanaka T, Taniguchi H. 2003. Proteomic analysis of rat liver peroxisome: Presence of peroxisome-specific isozyme of Lon protease. *J Biol Chem* **279:** (1) 421.
Kim SI, Kim JY, Kim EA, Kwon KH, Kim KW, Cho K, Lee JH, Nam MH, Yang DC, Yoo JS *et al*. 2003. Proteome analysis of hairy root from *Panax ginseng* C.A. Meyer using peptide fingerprinting, internal sequencing and expressed sequence tag data. *Proteomics* **3:** (12) 2379.
Kim ST, Cho KS, Yu S, Kim SG, Hong JC, Han CD, Bae DW, Myung AE, Kang KY. 2003. Proteomic analysis of differentially expressed proteins induced by rice blast fungus and elicitor in suspension-cultured rice cells. *Proteomics* **3:** (12) 2368.
Lee DY, Park YC, Kim HJ, Ryu YW, Seo JH. 2003. Proteomic analysis of *Candida magnoliae* strains by two-dimensional gel electrophoresis and mass spectrometry. *Proteomics* **3:** (12) 2330.
Lee EG, Kim JH, Shin YS, Shin GW, Suh MD, Kim DY, Kim YH, Kim GS, Jung TS. 2003. Establishment of a two-dimensional electrophoresis map for *Neospora caninum* tachyzoites by proteomics. *Proteomics* **3:** (12) 2339.
Lee PS, Lee KH. 2003. *Escherichia coli*: A model system that benefits from and contributes to the evolution of proteomics. *Biotechnol Bioeng* **84:** (7) 801.
Li KW, Hornshaw MP, Van der Schors RC, Watson R, Tate S, Casetta B, Jimenez CR, Gouwenberg Y, Gundelfinger ED, Smalla KH *et al*. 2004. Proteomics analysis of rat brain postsynaptic density - Implications of the diverse protein functional groups for the integration of synaptic physiology. *J Biol Chem* **279:** (2) 987.
Mihoub F, Mistou MY, Guillot A, Leveau JY, Boubetra A, Billaux F. 2003. Cold adaptation of *Escherichia coli*: Microbiological and proteomic approaches. *Int J Food Microbiol* **89:** (2-3) 171.
Nanjo Y, Asatsuma S, Itoh K, Hori H, Mitsui T. 2004. Proteomic identification of α-amylase isoforms encoded by *RAmy3B/3C* from germinating rice seeds. *Biosci Biotechnol Biochem* **68:** (1) 112.
Noel-Georis I, Vallaeys T, Chauvaux R, Monchy S, Falmagne R, Mergeay M, Wattiez R. 2004. Global analysis of the *Ralstonia metallidurans* proteome: Prelude for the large-scale study of heavy metal response. *Proteomics* **4:** (1) 151.
Ohlmeier S, Kastaniotis AJ, Hiltunen JK, Bergmann U. 2004. The yeast mitochondrial proteome, a study of fermentative and respiratory growth. *J Biol Chem* **279:** (6) 3956.
Pe'er I, Felder CE, Man O, Silman I, Sussman JL, Beckmann JS. 2004. Proteomic signatures: Amino acid and oligopeptide compositions differentiate among phyla. *Protein-Struct Funct Genet* **54:** (1) 20.
Pennington K, Mcgregor E, Beasley CL, Everall I, Cotter D, Dunn MJ. 2004. Optimization of the first dimension for separation by two-dimensional gel electrophoresis of basic proteins from human brain tissue. *Proteomics* **4:** (1) 27.
Pyo J, Hwang SI, Oh J, Lee SJ, Kang SJ, Kim JS, Lim J. 2003. Characterization of a bovine pregnancy associated protein using two-dimensional gel electrophoresis, N-terminal sequencing and mass spectrometry. *Proteomics* **3:** (12) 2420.
Sarry JE, Sommerer N, Sauvage FX, Bergoin A, Rossignol M, Albagnac G, Romieu C. 2004. Grape berry biochemistry revisited upon proteomic analysis of the mesocarp. *Proteomics* **4:** (1) 201.
Sender U, Bandow J, Engelmann S, Lindequist U, Hecker M. 2004. Proteomic signatures for daunomycin and adriamycin in *Bacillus subtilis*. *Pharmazie* **59:** (1) 65.
Tanaka N, Konishi H, Khan MMK, Komatsu S. 2004. Proteome analysis of rice tissues by two-dimensional electrophoresis: An approach to the investigation of gibberellin regulated proteins. *Mol Genet Genomics* **270:** (6) 485.
Ventelon-Debout M, Delalande F, Brizard JP, Diemer H, Van Dorsselaer A, Brugidou C. 2004. Proteome analysis of cultivar-specific deregulations of *Oryza sativa indica* and *O. sativa japonica* cellular suspensions undergoing rice yellow mottle virus infection. *Proteomics* **4:** (1) 216.
Vierstraete E, Verleyen P, Baggerman G, D'Hertog W, Van den Bergh G, Arckens L, De Loof A, Schoofs L. 2004. A proteomic approach for *the analysis of instantly released wound and immune proteins in Drosophila melanogaster* hemolymph. *Proc Natl Acad Sci U S A* **101:** (2) 470.
Wang JQ, Xue YF, Feng XL, Li XL, Wang H, Li W, Zhao CF, Cheng XJ, Ma YH, Zhou PJ *et al*. 2004. An analysis of the proteomic profile for *Thermoanaerobacter tengcongensis* under optimal culture conditions. *Proteomics* **4:** (1) 136.
Weeks ME, James DC, Robinson GK, Smales CM. 2004. Global changes in gene expression observed at the transition from growth to stationary phase in *Listeria monocytogenes* ScottA batch culture. *Proteomics* **4:** (1) 123.
Wiemann S, Mehrle A, Bechtel S, Wellenreuther R, Pepperkok R, Poustka A. 2003. cDNAs for functional genomics and proteomics: The German Consortium. *C R Biol* **326:** (10-11) 1003.
Yoshimura Y, Yamauchi Y, Shinkawa T, Taoka M, Donai H, Takahashi N, Isobe T, Yamauchi T. 2004. Molecular constituents of the postsynaptic density fraction revealed by proteomic analysis using multidimensional liquid chromatography-tandem mass spectrometry. *J Neurochem* **88:** (3) 759.
Yu JH, Yun SY, Lim JW, Kim H, Kim KH. 2003. Mass spectrometry and tandem mass spectrometry analysis of rat mitochondrial ATP synthase: Up-regulation in pancreatic acinar cells treated with cerulein. *Proteomics* **3:** (12) 2437.
Zhang B, Su YP, Ai GP, Liu XH, Wang FC, Cheng TM. 2003. Differentially expressed proteins of γ-ray irradiated mouse intestinal epithelial cells by two-dimensional electrophoresis and MALDI-TOF mass spectrometry. *World J Gastroenterol* **9:** (12) 2726.
Zhang KL, Yau PM, Chandrasekhar B, New R, Kondrat R, Imai BS, Bradbury ME. 2004. Differentiation between peptides containing acetylated or tri-methylated lysines by mass spectrometry: An application for determining lysine 9 acetylation and methylation of histone H3. *Proteomics* **4:** (1) 1.

### I2 Protein structural genomics

Goh CS, Lan N, Douglas SM, Wu BL, Echols N, Smith A, Milburn D, Montelione GT, Zhao HY, Gerstein M. 2004. Mining the structural genomics pipeline: Identification of protein properties that affect high-throughput experimental analysis. *J Mol Biol* **336:** (1) 115.
Ippel JH, Pouvreau L, Kroef T, Gruppen H, Versteeg G, Van den Putten P, Struik PC, van Mierlo CPM. 2004. *In vivo* uniform $^{15}$N-isotope labelling of plants: Using the greenhouse for structural proteomics. *Proteomics* **4:** (1) 226.
Liao L, Noble WS. 2003. Combining pairwise-sequence similarity and support vector machines for detecting remote protein evolutionary and structural relationships. *J Comput Biol* **10:** (6) 857.

### I3 Metabolomics

Bro C, Regenberg B, Nielsen J. 2004. Genome-wide transcriptional response of a *Saccharomyces cerevisiae* strain with an altered redox metabolism. *Biotechnol Bioeng* **85:** (3) 269.
Hellerstein MK. 2004. New stable isotope-mass spectrometric techniques for measuring fluxes through intact metabolic pathways in mammalian systems: Introduction of moving pictures into functional genomics and biochemical phenotyping. *Metab Eng* **6:** (1) 85.
Kim SI, Song SY, Kim KW, Ho EM, Oh KH. 2003. Proteomic analysis of the benzoate degradation pathway in *Acinetobacter* sp KS-1. *Res Microbiol* **154:** (10) 697.
Korke R, Gatti MD, Lau ALY, Lim JWE, Seow TK, Chung MCM, Hu WS. 2004. Large scale gene expression profiling of metabolic shift of mammalian cells in culture. *J Biotechnol* **107:** (1) 1.
Mahadevan R, Schilling CH. 2003. The effects of alternate optimal solutions in constraint-based genome-scale metabolic models. *Metab Eng* **5:** (4) 264.
Soo EC, Aubry AJ, Logan SM, Guerry P, Kelly JF, Young NM, Thibault P. 2004. Selective detection and identification of sugar nucleotides by CE-electrospray-MS and its application to bacterial

Copyright © 2004 John Wiley & Sons, Ltd. 

metabolomics. *Anal Chem* **76:** (3) 619.
Strauss AW. 2004. Tandem mass spectrometry in discovery of disorders of the metabolome. *J Clin Invest* **113:** (3) 354.
Tsoka S, Ouzounis CA. 2003. Metabolic database systems for the analysis of genome-wide function. *Biotechnol Bioeng* **84:** (7) 750.
Weckwerth W, Wenzel K, Fiehn O. 2004. Process for the integrated extraction identification, and quantification of metabolites, proteins and RNA to reveal their co-regulation in biochemical networks. *Proteomics* **4:** (1) 78.

### I4 Genomic approaches to development

Clarkson RWE, Watson CJ. 2003. Microarray analysis of the involution switch. *J Mammary Gland Biol Neoplasia* **8:** (3) 309.
Huang LY, Li BX, Luo C, Xie JY, Chen P, Liang SP. 2004. Proteome comparative analysis of gynogenetic haploid and diploid embryos of goldfish (*Carassius auratus*). *Proteomics* **4:** (1) 235.
Jin SH, Cho EH, Kol JE, Jung EH, Ahn B, Hahm JR, Kim JW, Kim CW, Kim DR. 2003. Comparative, analysis of nuclear proteins of B cells in different developmental stages. *Proteomics* **3:** (12) 2428.
Kamada T, Nito K, Hayashi H, Mano S, Hayashi M, Nishimura M. 2003. Functional differentiation of peroxisomes revealed by expression profiles of peroxisomal genes in *Arabidopsis thaliana*. *Plant Cell Physiol* **44:** (12) 1275.
Kim YO, Park SJ, Balaban RS, Nirenberg M, Kim Y. 2004. A functional genomic screen for cardiogenic genes using RNA interference in developing *Drosophila* embryos. *Proc Natl Acad Sci U S A* **101:** (1) 159.
Kury P, Abankwa D, Kruse F, Greiner-Petter R, Muller HW. 2004. Gene expression profiling reveals multiple novel intrinsic and extrinsic factors associated with axonal regeneration failure. *Eur J Neurosci* **19:** (1) 32.
McMullen CA, Andrade FH, Stahl JS. 2004. Functional and genomic changes in the mouse ocular motor system in response to light deprivation from birth. *J Neurosci* **24:** (1) 161.
Ng YY, van Kessel B, Lokhorst HM, Baert MRM, Van den Burg CMM, Bloem AC, Staal FJT. 2004. Gene-expression profiling of $CD34^+$ cells from various hematopoietic stem-cell sources reveals functional differences in stem-cell activity. *J Leukoc Biol* **75:** (2) 314.
Pollet N, Delius H, Niehrs C. 2003. *In situ* analysis of gene expression in *Xenopus* embryos. *C R Biol* **326:** (10-11) 1011.
Reinke V, Gil IS, Ward S, Kazmer K. 2004. Genome-wide germline-enriched and sex-biased expression profiles in *Caenorhabditis elegans*. *Development* **131:** (2) 311.
Rudolph MC, McManaman JL, Hunter L, Phang T, Neville MC. 2003. Functional development of the mammary gland: Use of expression profiling and trajectory clustering to reveal changes in gene expression during pregnancy, lactation, and involution. *J Mammary Gland Biol Neoplasia* **8:** (3) 287.
Srinivasan P, Abraham EG, Ghosh AK, Valenzuela J, Ribeiro JMC, Dimopoulos G, Kafatos FC, Adams JH, Fujioka H, Jacobs-Lorena M. 2004. Analysis of the *Plasmodium* and *Anopheles* transcriptomes during oocyst differentiation. *J Biol Chem* **279:** (7) 5581.
Strothmann K, Simoni M, Mathur P, Siakhamary S, Nieschlag E, Gromoll J. 2004. Gene expression profiling of mouse Sertoli cell lines. *Cell Tissue Res* **315:** (2) 249.
Symula DJ, Zhu YW, Schimenti JC, Rubin EM. 2004. Functional annotation of mouse mutations in embryonic stem cells by use of expression profiling. *Mamm Genome* **15:** (1) 1.
Ujino-Ihara T, Taguchi Y, Yoshimura K, Tsumura Y. 2003. Analysis of expressed sequence tags derived from developing seed and pollen cones of *Cryptomeria japonica*. *Plant Biol* **5:** (6) 600.
Wahl M, Shukunami C, Heinzmann U, Hamajima K, Hiraki YJ, Imai KJ. 2004. Transcriptome analysis of early chondrogenesis in ATDC5 cells induced by bone morphogenetic protein 4. *Genomics* **83:** (1) 45.
Zhao P, Seo JW, Wang ZY, Wang Y, Shneiderman B, Hoffman EP. 2003. *In vivo* filtering of *in vitro* expression data reveals MyoD targets. *C R Biol* **326:** (10-11) 1049.

### I5 Technological advances

Belbin TJ, Gaspar J, Haigentz M, Perez-Soler R, Keller SM, Prystowsky MB, Childs G, Socci ND. 2004. Indirect measurements of differential gene expression with cDNA microarrays. *Biotechniques* **36:** (2) 310.
Braun P, LaBaer J. 2004. High throughput protein production for functional proteomics. *Drug Discov Today* **9:** (Suppl 2) S1.
Brown SJ, Kuhn D, Wisser R, Power E, Schnell R. 2004. Quantification of sources of variation and accuracy of sequence discrimination in a replicated microarray experiment. *Biotechniques* **36:** (2) 324.
Chung YJ, Jonkers J, Kitson H, Fiegler H, Humphray S, Scott C, Hunt S, Yu Y, Nishijima I, Velds A *et al*. 2004. A whole-genome mouse BAC microarray with 1-Mb resolution for analysis of DNA copy number changes by array comparative genomic hybridization. *Genome Res* **14:** (1) 188.
Jiang L, He L, Fountoulakis M. 2004. Comparison of protein precipitation methods for sample preparation prior to proteomic analysis. *J Chromatogr A* **1023:** (2) 317.
Kim ST, Kim HS, Kim HJ, Kim SG, Kang SY, Lim DB, Kang KY. 2003. Prefractionation of protein samples for proteome analysis by sodium dodecyl sulfate-polyacrylamide gel electrophoresis. *Mol Cells* **16:** (3) 316.
Lee Y, Lee EK, Cho YW, Matsui T, Kang IC, Kim TS, Han MH. 2003. ProteoChip: A highly sensitive protein microarray prepared by a novel method of protein, immobilization for application of protein-protein interaction studies. *Proteomics* **3:** (12) 2289.
Li JN, Adams L, Schwartz SM, Bumgamer RE. 2003. RNA amplification, fidelity and reproducibility of expression profiling. *C R Biol* **326:** (10-11) 1021.
Lue RYP, Chen GYJ, Hu Y, Zhu Q, Yao SQ. 2004. Versatile protein biotinylation strategies for potential high-throughput proteomics. *J Am Chem Soc* **126:** (4) 1055.
Mackintosh JA, Choi HY, Bae SH, Veal DA, Bell PJ, Ferrari BC, Van Dyk DD, Verrills NM, Paik YK, Karuso P. 2003. A fluorescent natural product for ultra sensitive detection of proteins in one-dimensional and two-dimensional gel electrophoresis. *Proteomics* **3:** (12) 2273.
Marzolf B, Johnson MH. 2004. Validation of microarray image analysis accuracy. *Biotechniques* **36:** (2) 304.
Miyazaki K, Tsugita A. 2004. C-terminal sequencing method for peptides and proteins by the reaction with a vapor of perfluoric acid in acetic anhydride. *Proteomics* **4:** (1) 11.
Perelman S, Mazzella MA, Muschietti J, Zhu T, Casal JJ. 2003. Finding unexpected patterns in microarray data. *Plant Physiol* **133:** (4) 1717.
Rosati B, Grau F, Kuehler A, Rodriguez S, McKinnon D. 2004. Comparison of different probe-level analysis techniques for oligonucleotide microarrays. *Biotechniques* **36:** (2) 316.
Schramm A, Apostolov O, Sitek B, Pfeiffer K, Stuhler K, Meyer HE, Havers W, Eggert A. 2003. Proteomics: Techniques and applications in cancer research (German, English Abstract). *Klin Padiatr* **215:** (6) 293.
Sechi S, Oda Y. 2004. Quantitative proteomics using mass spectrometry. *Drug Discov Today* **9:** (Suppl 2) S41.
Shen Y, Tolic N, Masselon C, Pasa-Tolic L, Camp DG, Hixson KK, Zhao R, Anderson GA, Smith RD. 2004. Ultrasensitive proteomics using high-efficiency on-line micro-SPE-nanoLC-nanoESI MS and MS/MS. *Anal Chem* **76:** (1) 144.
Stanssens P, Zabeau M, Meersseman G, Remes G, Gansemans Y, Storm N, Hartmer R, Honisch C, Rodi CP, Bocker S *et al*. 2004. High-throughput MALDI-TOF discovery of genomic sequence polymorphisms. *Genome Res* **14:** (1) 126.
Tsai CA, Hsueh HM, Chen JJ. 2003. Estimation of false discovery rates in multiple testing: Application to gene microarray data. *Biometrics* **59:** (4) 1071.
Wang YJ. 2004. Immunostaining with dissociable antibody microarrays. *Proteomics* **4:** (1) 20.
Xue Y, Haas SA, Brino L, Gusnanto A, Reimers M, Talibi D, Vingron M, Ekwall K, Wright APH. 2004. A DNA microarray for fission yeast: Minimal changes in global gene expression after temperature shift. *Yeast* **21:** (1) 25.
Yan YL, Marriott G. 2004. Analysis of protein interactions using fluorescence technologies. *Drug Discov Today* **9:** (Suppl 2) S27.
Yu LR, Issaq HJ, Conrads TP, Uo T, Blonder J, Janini GM, Morrison RS, Veenstra TD. 2003. Evaluation of liquid chromatography-mass spectrometry for routine proteome analyses. *J Liq Chromatogr Relat Technol* **26:** (20) 3331.
Zheng LX, Liu J, Batalov S, Zhou DM, Orth A, Ding S, Schultz PG. 2004. An approach to genomewide screens of expressed small interfering RNAs in mammalian cells. *Proc Nat Acad Sci U S A* **101:** (1) 135.

Copyright © 2004 John Wiley & Sons, Ltd. 

### I6 Bioinformatics

Barker D. 2004. LVB: Parsimony and simulated annealing in the search for phylogenetic trees. *Bioinformatics* **20:** (2) 274.

Bellgard M, Hunter A, Kenworthy W. 2003. Microarray analysis using bioinformatics analysis audit trails (BAATs). *C R Biol* **326:** (10-11) 1083.

Benito M, Parker J, Du Q, Wu JY, Xang D, Perou CM, Marron JS. 2004. Adjustment of systematic microarray data biases. *Bioinformatics* **20:** (1) 105.

Bluthgen N, Kielbasa SM, Cajavec B, Herzel H. 2004. *HomGL* - comparing genelists across species and with different accession numbers. *Bioinformatics* **20:** (1) 125.

Braga-Neto U, Hashimoto R, Dougherty ER, Nguyen DV, Carroll RJ. 2004. Is cross-validation better than resubstitution for ranking genes? *Bioinformatics* **20:** (2) 253.

Brodie R, Roper RL, Upton C. 2004. JDotter: A Java interface to multiple dotplots generated by dotter. *Bioinformatics* **20:** (2) 279.

Burger A, Davidson D, Baldock R. 2004. Formalization of mouse embryo anatomy. *Bioinformatics* **20:** (2) 259.

Chapman BA, Bowers JE, Schulze SR, Paterson AH. 2004. A comparative phylogenetic approach for dating whole genome duplication events. *Bioinformatics* **20:** (2) 180.

Chiang JH, Yu HC, Hsu HJ. 2004. GIS: A biomedical text-mining system for gene information discovery. *Bioinformatics* **20:** (1) 120.

Crass T, Antes I, Basekow R, Bork P, Buning C, Christensen M, Claussen H, Ebeling C, Ernst P, Gailus-Durner V *et al*. 2004. The Helmholtz Network for Bioinformatics: An integrative web portal for bioinformatics resources. *Bioinformatics* **20:** (2) 268.

Dalla E, Verardo R, Lazarevic D, Marchionni L, Reid JF, Bahar N, Klaric E, Marcuzzi G, Marzio R, Belgrano A *et al*. 2003. LNCIB human full-length cDNAs collection: Towards a better comprehension of the human transcriptome. *C R Biol* **326:** (10-11) 967.

Darling AE, Mau B, Blattner FR, Perna NT. 2004. GRIL: Genome rearrangement and inversion locator. *Bioinformatics* **20:** (1) 122.

Datta S, Satten GA, Xia JZ, Heslin MJ, Datta S. 2004. An empirical Bayes adjustment to increase the sensitivity of detecting differentially expressed genes in microarray experiments. *Bioinformatics* **20:** (2) 235.

Deng MH, Zhang K, Mehta S, Chen T, Sun FZ. 2003. Prediction of protein function using protein-protein interaction data. *J Comput Biol* **10:** (6) 947.

Emrich SJ, Aluru S, Fu Y, Wen TJ, Narayanan M, Guo L, Ashlock DA, Schnable PS. 2004. A strategy for assembling the maize (*Zea mays* L.) genome. *Bioinformatics* **20:** (2) 140.

Goeman JJ, Van de Geer SA, De Kort F, Van Houwelingen HC. 2004. A global test for groups of genes: Testing association with a clinical outcome. *Bioinformatics* **20:** (1) 93.

Grant JD, Somers LA, Zhang Y, Manion FJ, Bidaut G, Ochs MF. 2004. FGDP: Functional genomics data pipeline for automated, multiple microarray data analyses. *Bioinformatics* **20:** (2) 282.

Gunaratne PH, Wu JQ, Garcia AM, Hulyk S, Worley KC, Margolin JF, Gibbs RA. 2003. Concatenation cDNA sequencing for transcriptome analysis. *C R Biol* **326:** (10-11) 971.

Hide W, Smedley D, McCarthy M, Kelso J. 2003. Application of eVOC: Controlled vocabularies for unifying gene expression data. *C R Biol* **326:** (10-11) 1089.

Hofacker IL, Priwitzer B, Stadler PF. 2004. Prediction of locally stable RNA secondary structures for genome-wide surveys. *Bioinformatics* **20:** (2) 186.

Huang SL, Wu LC, Liang HK, Pan KT, Horng JT, Ko MT. 2004. PGTdb: A database providing growth temperatures of prokaryotes. *Bioinformatics* **20:** (2) 276.

Huang Y, Li Y. 2004. Prediction of protein subcellular locations using fuzzy *k*-NN method. *Bioinformatics* **20:** (1) 21.

Ikeo K, Ishi-i J, Tamura T, Gojobori T, Tateno Y. 2003. CIBEX: Center for Information Biology gene EXpression database. *C R Biol* **326:** (10-11) 1079.

Juretic N, Bureau TE, Bruskiewich RM. 2004. Transposable element annotation of the rice genome. *Bioinformatics* **20:** (2) 155.

Kawaji H, Takenaka Y, Matsuda H. 2004. Graph-based clustering for finding distant relationships in a large set of protein sequences. *Bioinformatics* **20:** (2) 243.

Kerr MK. 2003. Linear models for microarray data analysis: Hidden similarities and differences. *J Comput Biol* **10:** (6) 891.

King RD, Whelan KE, Jones FM, Reiser PGK, Bryant CH, Muggleton SH, Kell DB, Oliver SG. 2004. Functional genomic hypothesis generation and experimentation by a robot scientist. *Nature* **427:** (697) 247.

Leader DP. 2004. BugView: A browser for comparing genomes. *Bioinformatics* **20:** (1) 129.

Lemke N, Heredia F, Barcellos CK, Dos Reis AN, Mombach JCM. 2004. Essentiality and damage in metabolic networks. *Bioinformatics* **20:** (1) 115.

Liu L, Gong G, Liu Y, Natarajan S, Larkin DM, Everts-van der Wind A, Rebeiz M, Beever JE. 2004. Multi-species comparative mapping *in silico* using the COMPASS strategy. *Bioinformatics* **20:** (2) 148.

Matukumalli LK, Grefenstette JJ, Sonstegard TS, Van Tassell CP. 2004. EST-PAGE - Managing and analyzing EST data. *Bioinformatics* **20:** (2) 286.

McGuffin LJ, Street S, Sorensen SA, Jones DT. 2004. The Genomic Threading Database. *Bioinformatics* **20:** (1) 131.

Melko OM, Mushegian AR. 2004. Distribution of words with a predefined range of mismatches to a DNA probe in bacterial genomes. *Bioinformatics* **20:** (1) 67.

Ogasawara O, Kawamoto S, Okubo K. 2003. Zipf's law and human transcriptomes: an explanation with an evolutionary model. *C R Biol* **326:** (10-11) 1097.

Orton RJ, Sellers WI, Gerloff DL. 2004. YETI: Yeast Exploration Tool Integrator. *Bioinformatics* **20:** (2) 284.

Perez AJ, Thode G, Trelles O. 2004. AnaGram: Protein function assignment. *Bioinformatics* **20:** (2) 291.

Prlic A, Domingues FS, Lackner P, Sippl MJ. 2004. WILMA - Automated annotation of protein sequences. *Bioinformatics* **20:** (1) 127.

Reymond N, Charles H, Duret L, Calevro F, Beslon G, Fayard JM. 2004. ROSO: Optimizing oligonucleotide probes for microarrays. *Bioinformatics* **20:** (2) 271.

Rocca-Serra P, Brazma A, Parkinson H, Sarkans U, Shojatalab M, Contrino S, Vilo J, Abeygunawardena N, Mukherjee G, Holloway E *et al*. 2003. ArrayExpress: A public database of gene expression data at EBI. *C R Biol* **326:** (10-11) 1075.

Segal MR, Dahlquist KD, Conklin BR. 2003. Regression approaches for microarray data analysis. *J Comput Biol* **10:** (6) 961.

Shibuya T, Kashima H, Konagaya A. 2004. Efficient filtering methods for clustering cDNAs with spliced sequence alignment. *Bioinformatics* **20:** (1) 29.

Shmueli O, Horn-Saban S, Chalifa-Caspi V, Shmoish M, Ophir R, Benjamin-Rodrig H, Safran M, Domany E, Lancet D. 2003. GeneNote: whole genome expression profiles in normal human tissues. *C R Biol* **326:** (10-11) 1067.

Sjolander K. 2004. Phylogenomic inference of protein molecular function: advances and challenges. *Bioinformatics* **20:** (2) 170.

Tu Q, Tang HX, Ding DF. 2004. MedBlast: Searching articles related to a biological sequence. *Bioinformatics* **20:** (1) 75.

Vasudeva K, Bhalla US. 2004. Adaptive stochastic-deterministic chemical kinetic simulations. *Bioinformatics* **20:** (1) 78.

Wang JH, Caron C, Mistou MY, Gitton C, Trubuil A. 2004. PARIS: A proteomic analysis and resources indexation system. *Bioinformatics* **20:** (1) 133.

Wang S, Ethier S. 2004. A generalized likelihood ratio test to identify differentially expressed genes from microarray data. *Bioinformatics* **20:** (1) 100.

Wichert S, Fokianos K, Strimmer K. 2004. Identifying periodically expressed transcripts in microarray time series data. *Bioinformatics* **20:** (1) 5.

Wren JD, Garner HR. 2004. Shared relationship analysis: Ranking set cohesion and commonalities within a literature-derived relationship network. *Bioinformatics* **20:** (2) 191.

Zhang L, Zhang AD, Ramanathan M. 2004. VizStruct: Exploratory visualization for gene expression profiling. *Bioinformatics* **20:** (1) 85.

Zhang Y, Waterman MS. 2003. An eulerian path approach to global multiple alignment for DNA sequences. *J Comput Biol* **10:** (6) 803.

Zhu QS, Deng YP, Vanka P, Brown SJ, Muthukrishnan S, Kramer KJ. 2004. Computational identification of novel chitinase-like proteins in the *Drosophila melanogaster* genome. *Bioinformatics* **20:** (2) 161.

Copyright © 2004 John Wiley & Sons, Ltd. 

## References

[PDF_00247] Agrawal Deepak, Chen Tingan, Irby Rosalyn, Quackenbush John, Chambers Ann F., Szabo Marianna, Cantor Alan, Coppola Domenico, Yeatman Timothy J. (2003). Osteopontin identified as colon cancer tumor progression marker.. C R Biol.

[PDF_00250] Ahn Woong Shick, Kim Ko-Woon, Bae Su Mi, Yoon Joo Hee, Lee Joon Mo, Namkoong Sung Eun, Kim Jin Hong, Kim Chong Kook, Lee Young Joo, Kim Yong-Wan (2003). Targeted cellular process profiling approach for uterine leiomyoma using cDNA microarray, proteomics and gene ontology analysis.. Int J Exp Pathol.

[PDF_00011] Auffray Charles, Imbeaud Sandrine, Roux-Rouquié Magali, Hood Leroy (2003). From functional genomics to systems biology: concepts and practices.. C R Biol.

[PDF_00637] Baggerman G., Clynen E., Huybrechts J., Verleyen P., Clerens S., De Loof A., Schoofs L. (2003). Peptide profiling of a single Locusta migratoria corpus cardiacum by nano-LC tandem mass spectrometry.. Peptides.

[PDF_00965] Barker D. (2004). LVB: parsimony and simulated annealing in the search for phylogenetic trees.. Bioinformatics.

[PDF_00233] Baud Sébastien, Vaultier Marie-Noëlle, Rochat Christine (2004). Structure and expression profile of the sucrose synthase multigene family in Arabidopsis.. J Exp Bot.

[PDF_00641] Baudouin-Cornu Peggy, Schuerer Katja, Marlière Philippe, Thomas Dominique (2003). Intimate evolution of proteins. Proteome atomic content correlates with genome base composition.. J Biol Chem.

[PDF_00886] Belbin Thomas J., Gaspar John, Haigentz Missak, Perez-Soler Roman, Keller Steven M., Prystowsky Michael B., Childs Geoffrey, Socci Nicholas D. (2004). Indirect measurements of differential gene expression with cDNA microarrays.. Biotechniques.

[PDF_00540] Belland Robert J., Nelson David E., Virok Dezso, Crane Deborah D., Hogan Daniel, Sturdevant Daniel, Beatty Wandy L., Caldwell Harlan D. (2003). Transcriptome analysis of chlamydial growth during IFN-gamma-mediated persistence and reactivation.. Proc Natl Acad Sci U S A.

[PDF_00967] Bellgard Matthew, Hunter Adam, Kenworthy William (2003). Microarray analysis using bioinformatics analysis audit trails (BAATs).. C R Biol.

[PDF_00970] Benito Monica, Parker Joel, Du Quan, Wu Junyuan, Xiang Dong, Perou Charles M., Marron J. S. (2004). Adjustment of systematic microarray data biases.. Bioinformatics.

[PDF_00017] Bertucci François, Loriod Béatrice, Nasser Valéry, Granjeaud Samuel, Tagett Rebecca, Braud Anne-Chantal, Patrice Viens, Houlgatte Rémi, Daniel Birnbaum, Nguyen Catherine (2003). Gene expression profiling of breast carcinomas using nylon DNA arrays.. C R Biol.

[PDF_00492] Biden Trevor J., Robinson Darren, Cordery Damien, Hughes William E., Busch Anna K. (2004). Chronic effects of fatty acids on pancreatic beta-cell function: new insights from functional genomics.. Diabetes.

[PDF_00973] Blüthgen Nils, Kiełbasa Szymon M., Cajavec Branka, Herzel Hanspeter (2004). HOMGL-comparing genelists across species and with different accession numbers.. Bioinformatics.

[PDF_00544] Bortoli Sylvie, Renault Valérie, Eveno Eric, Auffray Charles, Butler-Browne Gill, Piétu Geneviève (2003). Gene expression profiling of human satellite cells during muscular aging using cDNA arrays.. Gene.

[PDF_00495] Boutros Michael, Kiger Amy A., Armknecht Susan, Kerr Kim, Hild Marc, Koch Britta, Haas Stefan A., Paro Renato, Perrimon Norbert, Heidelberg Fly Array Consortium (2004). Genome-wide RNAi analysis of growth and viability in Drosophila cells.. Science.

[PDF_00976] Braga-Neto Ulisses, Hashimoto Ronaldo, Dougherty Edward R., Nguyen Danh V., Carroll Raymond J. (2004). Is cross-validation better than resubstitution for ranking genes?. Bioinformatics.

[PDF_00798] Bro Christoffer, Regenberg Birgitte, Nielsen Jens (2004). Genome-wide transcriptional response of a Saccharomyces cerevisiae strain with an altered redox metabolism.. Biotechnol Bioeng.

[PDF_00979] Brodie Ryan, Roper Rachel L., Upton Chris (2004). JDotter: a Java interface to multiple dotplots generated by dotter.. Bioinformatics.

[PDF_00891] Brown J. Steven, Kuhn David, Wisser Randall, Power Emilio, Schnell Raymond (2004). Quantification of sources of variation and accuracy of sequence discrimination in a replicated microarray experiment.. Biotechniques.

[PDF_00257] Bueno Raphael, Loughlin Kevin R., Powell Martha H., Gordon Gavin J. (2004). A diagnostic test for prostate cancer from gene expression profiling data.. J Urol.

[PDF_00649] Bunai Keigo, Ariga Masanori, Inoue Taro, Nozaki Manabu, Ogane Shinya, Kakeshita Hiroshi, Nemoto Tadashi, Nakanishi Hiroshi, Yamane Kunio (2004). Profiling and comprehensive expression analysis of ABC transporter solute-binding proteins of Bacillus subtilis membrane based on a proteomic approach.. Electrophoresis.

[PDF_00981] Burger Albert, Davidson Duncan, Baldock Richard (2004). Formalization of mouse embryo anatomy.. Bioinformatics.

[PDF_00027] Butterfield D. Allan, Boyd-Kimball Debra, Castegna Alessandra (2003). Proteomics in Alzheimer's disease: insights into potential mechanisms of neurodegeneration.. J Neurochem.

[PDF_00032] Carter Mark G., Piao Yulan, Dudekula Dawood B., Qian Yong, VanBuren Vincent, Sharov Alexei A., Tanaka Tetsuya S., Martin Patrick R., Bassey Uwem C., Stagg Carole A. (2003). The NIA cDNA project in mouse stem cells and early embryos.. C R Biol.

[PDF_00260] Cecconi Daniela, Astner Hubert, Donadelli Massimo, Palmieri Marta, Missiaglia Edoardo, Hamdan Mahmoud, Scarpa Aldo, Righetti Pier Giorgio (2003). Proteomic analysis of pancreatic ductal carcinoma cells treated with 5-aza-2'-deoxycytidine.. Electrophoresis.

[PDF_00654] Chan Leo Lai, Hodgkiss Ivor John, Lu Songhui, Lo Samuel Chun-Lap (2004). Use of two-dimensional gel electrophoresis proteome reference maps of dinoflagellates for species recognition of causative agents of harmful algal blooms.. Proteomics.

[PDF_00983] Chapman Brad A., Bowers John E., Schulze Stefan R., Paterson Andrew H. (2004). A comparative phylogenetic approach for dating whole genome duplication events.. Bioinformatics.

[PDF_00986] Chiang Jung-Hsien, Yu Hsu-Chun, Hsu Huai-Jen (2004). GIS: a biomedical text-mining system for gene information discovery.. Bioinformatics.

[PDF_00658] Cho Chang-Won, Lee So-Hee, Choi Jiyeon, Park Soo-Jin, Ha Dong-Jin, Kim Hyo-Jeong, Kim Chan-Wha (2003). Improvement of the two-dimensional gel electrophoresis analysis for the proteome study of Halobacterium salinarum.. Proteomics.

[PDF_00894] Chung Yeun-Jun, Jonkers Jos, Kitson Hannah, Fiegler Heike, Humphray Sean, Scott Carol, Hunt Sarah, Yu Yuejin, Nishijima Ichiko, Velds Arno (2004). A whole-genome mouse BAC microarray with 1-Mb resolution for analysis of DNA copy number changes by array comparative genomic hybridization.. Genome Res.

[PDF_00829] Clarkson Richard W. E., Watson Christine J. (2003). Microarray analysis of the involution switch.. J Mammary Gland Biol Neoplasia.

[PDF_00460] Connor E. E., Sonstegard T. S., Keele J. W., Bennett G. L., Williams J. L., Papworth R., Van Tassell C. P., Ashwell M. S. (2004). Physical and linkage mapping of mammary-derived expressed sequence tags in cattle.. Genomics.

[PDF_00036] Conrads Thomas P., Zhou Ming, Petricoin Emmanuel F., Liotta Lance, Veenstra Timothy D. (2003). Cancer diagnosis using proteomic patterns.. Expert Rev Mol Diagn.

[PDF_00661] Corpillo Davide, Gardini Giulia, Vaira Anna Maria, Basso Manuela, Aime Silvio, Accotto Gian Paolo, Fasano Mauro (2004). Proteomics as a tool to improve investigation of substantial equivalence in genetically modified organisms: the case of a virus-resistant tomato.. Proteomics.

[PDF_00988] Crass T., Antes I., Basekow R., Bork P., Buning C., Christensen M., Claussen H., Ebeling C., Ernst P., Gailus-Durner V. (2004). The Helmholtz Network for Bioinformatics: an integrative web portal for bioinformatics resources.. Bioinformatics.

[PDF_00273] Daibata Masanori, Matsuo Yoshinobu, Machida Hisanori, Taguchi Takahiro, Ohtsuki Yuji, Taguchi Hirokuni (2004). Differential gene-expression profiling in the leukemia cell lines derived from indolent and aggressive phases of CD56+ T-cell large granular lymphocyte leukemia.. Int J Cancer.

[PDF_00992] Dalla Emiliano, Verardo Roberto, Lazarević Dejan, Marchionni Luigi, Reid James F., Bahar Nabil, Klarić Enio, Marcuzzi Giacomo, Marzio Riccardo, Belgrano Anna (2003). LNCIB human full-length cDNAs collection: towards a better comprehension of the human transcriptome.. C R Biol.

[PDF_00996] Darling Aaron E., Mau Bob, Blattner Frederick R., Perna Nicole T. (2004). GRIL: genome rearrangement and inversion locator.. Bioinformatics.

[PDF_00998] Datta Susmita, Satten Glen A., Benos Dale J., Xia Jiazeng, Heslin Martin J., Datta Somnath (2004). An empirical bayes adjustment to increase the sensitivity of detecting differentially expressed genes in microarray experiments.. Bioinformatics.

[PDF_00039] De Angelis Maria, Gobbetti Marco (2004). Environmental stress responses in Lactobacillus: a review.. Proteomics.

[PDF_01002] Deng Minghua, Zhang Kui, Mehta Shipra, Chen Ting, Sun Fengzhu (2003). Prediction of protein function using protein-protein interaction data.. J Comput Biol.

[PDF_00498] Devlin Paul Francis, Yanovsky Marcelo Javier, Kay Steve A. (2003). A genomic analysis of the shade avoidance response in Arabidopsis.. Plant Physiol.

[PDF_00665] Dickinson Danielle N., La Duc Myron T., Haskins William E., Gornushkin Igor, Winefordner James D., Powell David H., Venkateswaran Kasthuri (2004). Species differentiation of a diverse suite of Bacillus spores by mass spectrometry-based protein profiling.. Appl Environ Microbiol.

[PDF_00041] Doolan D. L., Aguiar J. C., Weiss W. R., Sette A., Felgner P. L., Regis D. P., Quinones-Casas P., Yates J. R., Blair P. L., Richie T. L. (2003). Utilization of genomic sequence information to develop malaria vaccines.. J Exp Biol.

[PDF_00191] Doumith Michel, Cazalet Christel, Simoes Natalie, Frangeul Lionel, Jacquet Christine, Kunst Frank, Martin Paul, Cossart Pascale, Glaser Philippe, Buchrieser Carmen (2004). New aspects regarding evolution and virulence of Listeria monocytogenes revealed by comparative genomics and DNA arrays.. Infect Immun.

[PDF_00045] Dyrskjøt Lars (2003). Classification of bladder cancer by microarray expression profiling: towards a general clinical use of microarrays in cancer diagnostics.. Expert Rev Mol Diagn.

[PDF_00500] Eisenhaber Birgit, Wildpaner Michael, Schultz Carolyn J., Borner Georg H. H., Dupree Paul, Eisenhaber Frank (2003). Glycosylphosphatidylinositol lipid anchoring of plant proteins. Sensitive prediction from sequence- and genome-wide studies for Arabidopsis and rice.. Plant Physiol.

[PDF_01005] Emrich Scott J., Aluru Srinivas, Fu Yan, Wen Tsui-Jung, Narayanan Mahesh, Guo Ling, Ashlock Daniel A., Schnable Patrick S. (2004). A strategy for assembling the maize (Zea mays L.) genome.. Bioinformatics.

[PDF_00286] Fargiano Antonio A., Desai Kartiki V., Green Jeffrey E. (2003). Interrogating mouse mammary cancer models: insights from gene expression profiling.. J Mammary Gland Biol Neoplasia.

[PDF_00504] Gan Li, Ye Shiming, Chu Alan, Anton Kristin, Yi Saili, Vincent Valerie A., von Schack David, Chin Daniel, Murray Joseph, Lohr Scott (2003). Identification of cathepsin B as a mediator of neuronal death induced by Abeta-activated microglial cells using a functional genomics approach.. J Biol Chem.

[PDF_00061] Gass Sandra, Harris Jessica, Ormandy Chris, Brisken Cathrin (2003). Using gene expression arrays to elucidate transcriptional profiles underlying prolactin function.. J Mammary Gland Biol Neoplasia.

[PDF_00213] Ge Hong, Chuang Yao-Yu Eric, Zhao Shuping, Tong Min, Tsai Mong-Hsun, Temenak Joseph J., Richards Allen L., Ching Wei-Mei (2004). Comparative genomics of Rickettsia prowazekii Madrid E and Breinl strains.. J Bacteriol.

[PDF_00508] Giaever Guri, Flaherty Patrick, Kumm Jochen, Proctor Michael, Nislow Corey, Jaramillo Daniel F., Chu Angela M., Jordan Michael I., Arkin Adam P., Davis Ronald W. (2004). Chemogenomic profiling: identifying the functional interactions of small molecules in yeast.. Proc Natl Acad Sci U S A.

[PDF_00560] Gibbings J. George, Cook Brian P., Dufault Michael R., Madden Stephen L., Khuri Sawsan, Turnbull Chris J., Dunwell Jim M. (2003). Global transcript analysis of rice leaf and seed using SAGE technology.. Plant Biotechnol J.

[PDF_00289] Godl Klaus, Wissing Josef, Kurtenbach Alexander, Habenberger Peter, Blencke Stephanie, Gutbrod Heidrun, Salassidis Kostadinos, Stein-Gerlach Matthias, Missio Andrea, Cotten Matt (2003). An efficient proteomics method to identify the cellular targets of protein kinase inhibitors.. Proc Natl Acad Sci U S A.

[PDF_00785] Goh Chern-Sing, Lan Ning, Douglas Shawn M., Wu Baolin, Echols Nathaniel, Smith Andrew, Milburn Duncan, Montelione Gaetano T., Zhao Hongyu, Gerstein Mark (2004). Mining the structural genomics pipeline: identification of protein properties that affect high-throughput experimental analysis.. J Mol Biol.

[PDF_00064] Goldstein Adrian M. (2003). Changing paradigms in dermatology: proteomics: a new approach to skin disease.. Clin Dermatol.

[PDF_00066] Gott Jonatha M. (2003). Expanding genome capacity via RNA editing.. C R Biol.

[PDF_01011] Grant Jeffrey D., Somers Luke A., Zhang Yue, Manion Frank J., Bidaut Ghislain, Ochs Michael F. (2004). FGDP: functional genomics data pipeline for automated, multiple microarray data analyses.. Bioinformatics.

[PDF_00293] Green Jeffrey E., Desai Kartiki, Ye Yumei, Kavanaugh Claudine, Calvo Alfonso, Huh Jung-Im (2004). Genomic approaches to understanding mammary tumor progression in transgenic mice and responses to therapy.. Clin Cancer Res.

[PDF_00297] Greshock Joel, Naylor Tara L., Margolin Adam, Diskin Sharon, Cleaver Stephen H., Futreal P. Andrew, deJong Pieter J., Zhao Shaying, Liebman Michael, Weber Barbara L. (2003). 1-Mb resolution array-based comparative genomic hybridization using a BAC clone set optimized for cancer gene analysis.. Genome Res.

[PDF_00068] Gros François (2003). From the messenger RNA saga to the transcriptome era.. C R Biol.

[PDF_01014] Gunaratne Preethi H., Wu Jia Qian, Garcia Angela M., Hulyk Steven, Worley Kim C., Margolin Judith F., Gibbs Richard A. (2003). Concatenation cDNA sequencing for transcriptome analysis.. C R Biol.

[PDF_00073] Han Ze-Guang, Zhao Guo-Ping, Chen Zhu (2003). Transcriptome study in China.. C R Biol.

[PDF_00512] Hanke Guy Thomas, Kimata-Ariga Yoko, Taniguchi Isao, Hase Toshiharu (2003). A post genomic characterization of Arabidopsis ferredoxins.. Plant Physiol.

[PDF_00075] Hayashizaki Yoshihide (2003). The Riken mouse genome encyclopedia project.. C R Biol.

[PDF_00077] Hayes Finbarr (2003). Transposon-based strategies for microbial functional genomics and proteomics.. Annu Rev Genet.

[PDF_00301] He Qing-Yu, Chen Jia, Kung Hsiang-Fu, Yuen Anthony Po-Wing, Chiu Jen-Fu (2004). Identification of tumor-associated proteins in oral tongue squamous cell carcinoma by proteomics.. Proteomics.

[PDF_00675] Heazlewood Joshua L., Tonti-Filippini Julian S., Gout Alexander M., Day David A., Whelan James, Millar A. Harvey (2003). Experimental analysis of the Arabidopsis mitochondrial proteome highlights signaling and regulatory components, provides assessment of targeting prediction programs, and indicates plant-specific mitochondrial proteins.. Plant Cell.

[PDF_00079] Hegde Priti S., White Ian R., Debouck Christine (2003). Interplay of transcriptomics and proteomics.. Curr Opin Biotechnol.

[PDF_00083] Henshall Susan (2003). Tissue microarrays.. J Mammary Gland Biol Neoplasia.

[PDF_00563] Herath Chandana B., Shiojima Satoshi, Ishiwata Hiroko, Katsuma Susumu, Kadowaki Tadashi, Ushizawa Koichi, Imai Kei, Takahashi Toru, Hirasawa Akira, Tsujimoto Gozoh (2004). Pregnancy-associated changes in genome-wide gene expression profiles in the liver of cow throughout pregnancy.. Biochem Biophys Res Commun.

[PDF_00680] Herranen Mirkka, Battchikova Natalia, Zhang Pengpeng, Graf Alexander, Sirpiö Sari, Paakkarinen Virpi, Aro Eva-Mari (2004). Towards functional proteomics of membrane protein complexes in Synechocystis sp. PCC 6803.. Plant Physiol.

[PDF_01017] Hide Winston, Smedley Damian, McCarthy Mark, Kelso Janet (2003). Application of eVOC: controlled vocabularies for unifying gene expression data.. C R Biol.

[PDF_00568] Higgins Vincent J., Rogers Peter J., Dawes Ian W. (2003). Application of genome-wide expression analysis to identify molecular markers useful in monitoring industrial fermentations.. Appl Environ Microbiol.

[PDF_01020] Hofacker I. L., Priwitzer B., Stadler P. F. (2004). Prediction of locally stable RNA secondary structures for genome-wide surveys.. Bioinformatics.

[PDF_00831] Huang Lingyun, Li Bingxia, Luo Chen, Xie Jinyun, Chen Ping, Liang Songping (2004). Proteome comparative analysis of gynogenetic haploid and diploid embryos of goldfish (Carassius auratus).. Proteomics.

[PDF_01023] Huang Shir-Ly, Wu Li-Cheng, Liang Han-Kuen, Pan Kuan-Ting, Horng Jorng-Tzong, Ko Ming-Tat (2004). PGTdb: a database providing growth temperatures of prokaryotes.. Bioinformatics.

[PDF_00571] Hudson Matthew E., Quail Peter H. (2003). Identification of promoter motifs involved in the network of phytochrome A-regulated gene expression by combined analysis of genomic sequence and microarray data.. Plant Physiol.

[PDF_01028] Ikeo Kazuho, Ishi-i Jun, Tamura Takurou, Gojobori Takashi, Tateno Yoshio (2003). CIBEX: center for information biology gene expression database.. C R Biol.

[PDF_00789] Ippel Johannes H., Pouvreau Laurice, Kroef Toos, Gruppen Harry, Versteeg Geurt, van den Putten Peter, Struik Paul C., van Mierlo Carlo P. M. (2004). In vivo uniform (15)N-isotope labelling of plants: using the greenhouse for structural proteomics.. Proteomics.

[PDF_00216] Islas Sara, Becerra Arturo, Luisi P. Luigi, Lazcano Antonio (2004). Comparative genomics and the gene complement of a minimal cell.. Orig Life Evol Biosph.

[PDF_00304] Iwata Shin-ichi, Nomoto Masahiro, Morioka Hirofumi, Miyata Atsuro (2004). Gene expression profiling in the midbrain of striatal 6-hydroxydopamine-injected mice.. Synapse.

[PDF_00575] Jenson S. D., Robetorye R. S., Bohling S. D., Schumacher J. A., Morgan J. W., Lim M. S., Elenitoba-Johnson K. S. J. (2003). Validation of cDNA microarray gene expression data obtained from linearly amplified RNA.. Mol Pathol.

[PDF_00899] Jiang Lei, He Lin, Fountoulakis Michael (2004). Comparison of protein precipitation methods for sample preparation prior to proteomic analysis.. J Chromatogr A.

[PDF_00579] Jiao Yuling, Yang Hongjuan, Ma Ligeng, Sun Ning, Yu Haiyuan, Liu Tie, Gao Ying, Gu Hongya, Chen Zhangliang, Wada Masamitsu (2003). A genome-wide analysis of blue-light regulation of Arabidopsis transcription factor gene expression during seedling development.. Plant Physiol.

[PDF_00515] Jim Kam, Parmar Kush, Singh Mona, Tavazoie Saeed (2004). A cross-genomic approach for systematic mapping of phenotypic traits to genes.. Genome Res.

[PDF_00834] Jin Sun Hee, Cho Eun Hye, Ko Jung Eun, Jung Eun Hee, Ahn Byungchan, Hahm Jong Ryeal, Kim Jae Won, Kim Choong Won, Kim Deok Ryong (2003). Comparative analysis of nuclear proteins of B cells in different developmental stages.. Proteomics.

[PDF_00307] Joo Won-A, Kang Mee-Jeong, Son Won-kyu, Lee Hyun-Ju, Lee Do-Youn, Lee Eunil, Kim Chan-Wha (2003). Monitoring protein expression by proteomics: human plasma exposed to benzene.. Proteomics.

[PDF_01031] Juretic Nikoleta, Bureau Thomas E., Bruskiewich Richard M. (2004). Transposable element annotation of the rice genome.. Bioinformatics.

[PDF_00837] Kamada Tomoe, Nito Kazumasa, Hayashi Hiroshi, Mano Shoji, Hayashi Makoto, Nishimura Mikio (2003). Functional differentiation of peroxisomes revealed by expression profiles of peroxisomal genes in Arabidopsis thaliana.. Plant Cell Physiol.

[PDF_00310] Kang Hio Chung, Kim Il-Jin, Park Jae-Hyun, Shin Yong, Ku Ja-Lok, Jung Mi Sun, Yoo Byong Chul, Kim Hark Kyun, Park Jae-Gahb (2004). Identification of genes with differential expression in acquired drug-resistant gastric cancer cells using high-density oligonucleotide microarrays.. Clin Cancer Res.

[PDF_00587] Kao Katy C., Yang Young-Lyeol, Boscolo Riccardo, Sabatti Chiara, Roychowdhury Vwani, Liao James C. (2003). Transcriptome-based determination of multiple transcription regulator activities in Escherichia coli by using network component analysis.. Proc Natl Acad Sci U S A.

[PDF_00106] Kato Kikuya (2003). Adaptor-tagged competitive PCR: study of the mammalian nervous system.. C R Biol.

[PDF_01033] Kawaji Hideya, Takenaka Yoichi, Matsuda Hideo (2004). Graph-based clustering for finding distant relationships in a large set of protein sequences.. Bioinformatics.

[PDF_01036] Kerr M. Kathleen (2003). Linear models for microarray data analysis: hidden similarities and differences.. J Comput Biol.

[PDF_00314] Khawaja Xavier, Xu Jun, Liang Jin-Jun, Barrett James E. (2004). Proteomic analysis of protein changes developing in rat hippocampus after chronic antidepressant treatment: Implications for depressive disorders and future therapies.. J Neurosci Res.

[PDF_00684] Kikuchi Miki, Hatano Naoya, Yokota Sadaki, Shimozawa Nobuyuki, Imanaka Tsuneo, Taniguchi Hisaaki (2003). Proteomic analysis of rat liver peroxisome: presence of peroxisome-specific isozyme of Lon protease.. J Biol Chem.

[PDF_00318] Kim Chang-Hoon, Kim Do Kyun, Choi Seung Jin, Choi Kun Ho, Song Kyoung Seob, Chi Janghoon, Koo Ja Seok, Hwang Seung Yong, Yoon Joo-Heon, Seong Je Kyung (2003). Proteomic and transcriptomic analysis of interleukin-1beta treated lung carcinoma cell line.. Proteomics.

[PDF_00591] Kim Hyun J., Ishidou E., Kitagawa E., Momose Y., Iwahashi H. (2004). A yeast DNA microarray for the evaluation of toxicity in environmental water containing burned ash.. Environ Monit Assess.

[PDF_00368] Kim Okhee, Park Misun, Kang Hoil, Lim Sinae, Lee Choon-Taek (2003). Differential protein expressions induced by adenovirus-mediated p16 gene transfer into Balb/c nude mouse.. Proteomics.

[PDF_00688] Kim Seung Il, Kim Jin Young, Kim Eun A., Kwon Kyung-Hoon, Kim Kyung-Wook, Cho Kun, Lee Jeong Hwa, Nam Myung Hee, Yang Deok-Chun, Yoo Jong Shin (2003). Proteome analysis of hairy root from Panax ginseng C.A. Meyer using peptide fingerprinting, internal sequencing and expressed sequence tag data.. Proteomics.

[PDF_00805] Kim Seung Il, Song Seung Youl, Kim Kyung Wook, Ho Eun Mi, Oh Kye Heon (2003). Proteomic analysis of the benzoate degradation pathway in Acinetobacter sp. KS-1.. Res Microbiol.

[PDF_00594] Kim Seung-Ki, Wang Kyu-Chang, Hong Soo Jin, Chung Chun-Kee, Lim Su-Young, Kim Young-Yim, Chi Je G., Kim Chong Jai, Chung You-Nam, Kim Hyun Jib (2003). Gene expression profile analyses of cortical dysplasia by cDNA arrays.. Epilepsy Res.

[PDF_00692] Kim Sun Tae, Cho Kyu Seong, Yu Seok, Kim Sang Gon, Hong Jong Chan, Han Chang-deok, Bae Dong Won, Nam Myung Hee, Kang Kyu Young (2003). Proteomic analysis of differentially expressed proteins induced by rice blast fungus and elicitor in suspension-cultured rice cells.. Proteomics.

[PDF_00902] Kim Sun Tae, Kim Hyun Soo, Kim Han Ju, Kim Sang Gon, Kang Sun Young, Lim Dong Bin, Kang Kyu Young (2003). Prefractionation of protein samples for proteome analysis by sodium dodecyl sulfate-polyacrylamide gel electrophoresis.. Mol Cells.

[PDF_00841] Kim Yong-Ou, Park Sang-Joon, Balaban Robert S., Nirenberg Marshall, Kim Yongsok (2003). A functional genomic screen for cardiogenic genes using RNA interference in developing Drosophila embryos.. Proc Natl Acad Sci U S A.

[PDF_01038] King Ross D., Whelan Kenneth E., Jones Ffion M., Reiser Philip G. K., Bryant Christopher H., Muggleton Stephen H., Kell Douglas B., Oliver Stephen G. (2004). Functional genomic hypothesis generation and experimentation by a robot scientist.. Nature.

[PDF_00238] Kofuji Rumiko, Sumikawa Naomi, Yamasaki Misuzu, Kondo Kimihiko, Ueda Kunihiko, Ito Motomi, Hasebe Mitsuyasu (2003). Evolution and divergence of the MADS-box gene family based on genome-wide expression analyses.. Mol Biol Evol.

[PDF_00597] Kreps Joel, Budworth Paul, Goff Steve, Wang Ronglin (2003). Identification of putative plant cold responsive regulatory elements by gene expression profiling and a pattern enumeration algorithm.. Plant Biotechnol J.

[PDF_00464] Kuhl Joseph C., Cheung Foo, Yuan Qiaoping, Martin William, Zewdie Yayeh, McCallum John, Catanach Andrew, Rutherford Paul, Sink Kenneth C., Jenderek Maria (2003). A unique set of 11,008 onion expressed sequence tags reveals expressed sequence and genomic differences between the monocot orders Asparagales and Poales.. Plant Cell.

[PDF_00469] Kwon Kyung-Hoon, Kim Mioak, Kim Jin Young, Kim Kyung Wook, Kim Seung Il, Park Young Mok, Yoo Jong Shin (2003). Efficiency improvement of peptide identification for an organism without complete genome sequence, using expressed sequence tag database and tandem mass spectral data.. Proteomics.

[PDF_00322] Lapointe Jacques, Li Chunde, Higgins John P., van de Rijn Matt, Bair Eric, Montgomery Kelli, Ferrari Michelle, Egevad Lars, Rayford Walter, Bergerheim Ulf (2004). Gene expression profiling identifies clinically relevant subtypes of prostate cancer.. Proc Natl Acad Sci U S A.

[PDF_01041] Leader David P. (2004). BugView: a browser for comparing genomes.. Bioinformatics.

[PDF_00326] Lee Chih-Lei, Hsiao He-Hsuan, Lin Chia-Wei, Wu Szu-Pei, Huang Shiuan-Yi, Wu Chi-Yue, Wang Andrew H-J, Khoo Kay-Hooi (2003). Strategic shotgun proteomics approach for efficient construction of an expression map of targeted protein families in hepatoma cell lines.. Proteomics.

[PDF_00696] Lee Do-Yup, Park Yong-Cheol, Kim Hyo-Jin, Ryu Yeon-Woo, Seo Jin-Ho (2003). Proteomic analysis of Candida magnoliae strains by two-dimensional gel electrophoresis and mass spectrometry.. Proteomics.

[PDF_00699] Lee Eung Goo, Kim Jae Hoon, Shin Yong Seung, Shin Gee Wook, Suh Myung Deuk, Kim Dae Yong, Kim Yong Hwan, Kim Gon Sup, Jung Tae Sung (2003). Establishment of a two-dimensional electrophoresis map for Neospora caninum tachyzoites by proteomics.. Proteomics.

[PDF_00518] Lee Shinyoung, Kim Joonyul, Son Jun-Seock, Nam Jongmin, Jeong Dong-Hoon, Lee Keunsub, Jang Seonghoe, Yoo Jihye, Lee Jinwon, Lee Dong-Yeon (2003). Systematic reverse genetic screening of T-DNA tagged genes in rice for functional genomic analyses: MADS-box genes as a test case.. Plant Cell Physiol.

[PDF_00906] Lee Yoonsuk, Lee Eun Kyoung, Cho Yong Wan, Matsui Takuya, Kang In-Cheol, Kim Tai-Sun, Han Moon Hi (2003). ProteoChip: a highly sensitive protein microarray prepared by a novel method of protein immobilization for application of protein-protein interaction studies.. Proteomics.

[PDF_01043] Lemke Ney, Herédia Fabiana, Barcellos Cláudia K., Dos Reis Adriana N., Mombach José C. M. (2004). Essentiality and damage in metabolic networks.. Bioinformatics.

[PDF_00330] Leonard P., Sharp T., Henderson S., Hewitt D., Pringle J., Sandison A., Goodship A., Whelan J., Boshoff C. (2003). Gene expression array profile of human osteosarcoma.. Br J Cancer.

[PDF_00333] Li Ailing, Li Hongwei, Jin Gang, Xiu Ruijuan (2003). A proteomic study on cell cycle progression of endothelium exposed to tumor conditioned medium and the possible role of cyclin D1/E.. Clin Hemorheol Microcirc.

[PDF_00910] Li Jiangning, Adams Larry, Schwartz S. M., Bumgarner Roger E. (2003). RNA amplification, fidelity and reproducibility of expression profiling.. C R Biol.

[PDF_00706] Li Ka Wan, Hornshaw Martin P., Van Der Schors Roel C., Watson Rod, Tate Stephen, Casetta Bruno, Jimenez Connie R., Gouwenberg Yvonne, Gundelfinger Eckart D., Smalla Karl-Heinz (2003). Proteomics analysis of rat brain postsynaptic density. Implications of the diverse protein functional groups for the integration of synaptic physiology.. J Biol Chem.

[PDF_00337] Li Meihua, Lin Yu-Min, Hasegawa Suguru, Shimokawa Takashi, Murata Kohei, Kameyama Masao, Ishikawa Osamu, Katagiri Toyomasa, Tsunoda Tatsuhiko, Nakamura Yusuke (2004). Genes associated with liver metastasis of colon cancer, identified by genome-wide cDNA microarray.. Int J Oncol.

[PDF_00341] Li Qingsheng, Schacker Timothy, Carlis John, Beilman Gregory, Nguyen Phuong, Haase Ashley T. (2004). Functional genomic analysis of the response of HIV-1-infected lymphatic tissue to antiretroviral therapy.. J Infect Dis.

[PDF_00794] Liao Li, Noble William Stafford (2003). Combining pairwise sequence similarity and support vector machines for detecting remote protein evolutionary and structural relationships.. J Comput Biol.

[PDF_00344] Lilien Ryan H., Farid Hany, Donald Bruce R. (2003). Probabilistic disease classification of expression-dependent proteomic data from mass spectrometry of human serum.. J Comput Biol.

[PDF_00347] Liu Jing, Dehbi Mohammed, Moeck Greg, Arhin Francis, Bauda Pascale, Bergeron Dominique, Callejo Mario, Ferretti Vincent, Ha Nhuan, Kwan Tony (2004). Antimicrobial drug discovery through bacteriophage genomics.. Nat Biotechnol.

[PDF_01046] Liu Lei, Gong George, Liu Yong, Natarajan Shreedhar, Larkin Denis M., Everts-van der Wind Annelie, Rebeiz Mark, Beever Jonathan E. (2004). Multi-species comparative mapping in silico using the COMPASS strategy.. Bioinformatics.

[PDF_00116] Liu Ying, Sturley Stephen L. (2004). Nutritional genomics in yeast models.. Nutrition.

[PDF_00118] Lock Christopher B., Heller Renu A. (2003). Gene microarray analysis of multiple sclerosis lesions.. Trends Mol Med.

[PDF_00350] Loni Lucia, De Braud Filippo, Zinzani Pier Luigi, Danesi Romano (2003). Pharmacogenetics and proteomics of anticancer drugs in non-Hodgkin's lymphoma.. Leuk Lymphoma.

[PDF_00913] Lue Rina Y. P., Chen Grace Y. J., Hu Yi, Zhu Qing, Yao Shao Q. (2004). Versatile protein biotinylation strategies for potential high-throughput proteomics.. J Am Chem Soc.

[PDF_00353] Lum Pek Yee, Armour Christopher D., Stepaniants Sergey B., Cavet Guy, Wolf Maria K., Butler J. Scott, Hinshaw Jerald C., Garnier Philippe, Prestwich Glenn D., Leonardson Amy (2004). Discovering modes of action for therapeutic compounds using a genome-wide screen of yeast heterozygotes.. Cell.

[PDF_00916] Mackintosh James A., Choi Hung-Yoon, Bae Soo-Han, Veal Duncan A., Bell Philip J., Ferrari Belinda C., Van Dyk Derek D., Verrills Nicole M., Paik Young-Ki, Karuso Peter (2003). A fluorescent natural product for ultra sensitive detection of proteins in one-dimensional and two-dimensional gel electrophoresis.. Proteomics.

[PDF_00811] Mahadevan R., Schilling C. H. (2003). The effects of alternate optimal solutions in constraint-based genome-scale metabolic models.. Metab Eng.

[PDF_00357] Maitra Anirban, Hansel Donna E., Argani Pedram, Ashfaq Raheela, Rahman Ayman, Naji Ali, Deng Shaoping, Geradts Joseph, Hawthorne LesleyAnn, House Michael G. (2003). Global expression analysis of well-differentiated pancreatic endocrine neoplasms using oligonucleotide microarrays.. Clin Cancer Res.

[PDF_00361] Martin Daniel B., Gifford David R., Wright Michael E., Keller Andrew, Yi Eugene, Goodlett David R., Aebersold Reudi, Nelson Peter S. (2004). Quantitative proteomic analysis of proteins released by neoplastic prostate epithelium.. Cancer Res.

[PDF_00920] Marzolf Bruz, Johnson Michael H. (2004). Validation of microarray image analysis accuracy.. Biotechniques.

[PDF_01049] Matukumalli Lakshmi K., Grefenstette John J., Sonstegard Tad S., Van Tassell Curtis P. (2004). EST-PAGE--managing and analyzing EST data.. Bioinformatics.

[PDF_00219] McCarroll Steven A., Murphy Coleen T., Zou Sige, Pletcher Scott D., Chin Chen-Shan, Jan Yuh Nung, Kenyon Cynthia, Bargmann Cornelia I., Li Hao (2004). Comparing genomic expression patterns across species identifies shared transcriptional profile in aging.. Nat Genet.

[PDF_01052] McGuffin Liam J., Street Stefano, Sørensen Søren-Aksel, Jones David T. (2004). The genomic threading database.. Bioinformatics.

[PDF_00848] McMullen Colleen A., Andrade Francisco H., Stahl John S. (2004). Functional and genomic changes in the mouse ocular motor system in response to light deprivation from birth.. J Neurosci.

[PDF_00122] McShane Lisa M., Shih Joanna H., Michalowska Aleksandra M. (2003). Statistical issues in the design and analysis of gene expression microarray studies of animal models.. J Mammary Gland Biol Neoplasia.

[PDF_00711] Mihoub Fatma, Mistou Michel-Yves, Guillot Alain, Leveau Jean-Yves, Boubetra Abdelkader, Billaux François (2003). Cold adaptation of Escherichia coli: microbiological and proteomic approaches.. Int J Food Microbiol.

[PDF_00365] Miller Ingrid, Teinfalt Monika, Leschnik Michael, Wait Robin, Gemeiner Manfred (2004). Nonreducing two-dimensional gel electrophoresis for the detection of Bence Jones proteins in serum and urine.. Proteomics.

[PDF_00127] Minamikawa-Tachino Reiko, Kabuyama Noriko, Gotoh Toshiyuki, Kagei Seiichiro, Naruse Masatoshi, Kisu Yasutomo, Togashi Takushi, Sugano Sumio, Usami Hitohide, Nomura Nobuo (2003). High-throughput classification of images of cells transfected with cDNA clones.. C R Biol.

[PDF_00131] Mobasheri A., Airley R., Foster C. S., Schulze-Tanzil G., Shakibaei M. (2004). Post-genomic applications of tissue microarrays: basic research, prognostic oncology, clinical genomics and drug discovery.. Histol Histopathol.

[PDF_00138] Nagase Takahiro, Kikuno Reiko, Ohara Osamu (2003). The Kazusa cDNA project for identification of unknown human transcripts.. C R Biol.

[PDF_00473] Nam Myung Hee, Heo Eun Joo, Kim Jin Young, Kim Seung Il, Kwon Kyung-Hoon, Seo Jong Bok, Kwon Ohoak, Yoo Jong Shin, Park Young Mok (2003). Proteome analysis of the responses of Panax ginseng C. A. Meyer leaves to high light: use of electrospray ionization quadrupole-time of flight mass spectrometry and expressed sequence tag data.. Proteomics.

[PDF_00714] Nanjo Yohei, Asatsuma Satoru, Itoh Kimiko, Hori Hidetaka, Mitsui Toshiaki (2004). Proteomic identification of alpha-amylase isoforms encoded by RAmy3B/3C from germinating rice seeds.. Biosci Biotechnol Biochem.

[PDF_00604] Narasimhan Sukanya, Caimano Melissa J., Liang Fang Ting, Santiago Felix, Laskowski Michelle, Philipp Mario T., Pachner Andrew R., Radolf Justin D., Fikrig Erol, Camaino Melissa J. (2003). Borrelia burgdorferi transcriptome in the central nervous system of non-human primates.. Proc Natl Acad Sci U S A.

[PDF_00851] Ng Yuk Yin, van Kessel Berris, Lokhorst Henk M., Baert Miranda R. M., van den Burg Caroline M. M., Bloem Andries C., Staal Frank J. T. (2003). Gene-expression profiling of CD34+ cells from various hematopoietic stem-cell sources reveals functional differences in stem-cell activity.. J Leukoc Biol.

[PDF_00478] Nobis William, Ren Xiaoning, Suchyta Steven P., Suchyta Thomas R., Zanella Adroaldo J., Coussens Paul M. (2003). Development of a porcine brain cDNA library, EST database, and microarray resource.. Physiol Genomics.

[PDF_00717] Noël-Georis Isabelle, Vallaeys Tatiana, Chauvaux Renaud, Monchy Sébastien, Falmagne Paul, Mergeay Max, Wattiez Ruddy (2004). Global analysis of the Ralstonia metallidurans proteome: prelude for the large-scale study of heavy metal response.. Proteomics.

[PDF_00608] O'Rourke Sean M., Herskowitz Ira (2003). Unique and redundant roles for HOG MAPK pathway components as revealed by whole-genome expression analysis.. Mol Biol Cell.

[PDF_01057] Ogasawara Osamu, Kawamoto Shoko, Okubo Kousaku (2003). Zipf's law and human transcriptomes: an explanation with an evolutionary model.. C R Biol.

[PDF_00721] Ohlmeier Steffen, Kastaniotis Alexander J., Hiltunen J. Kalervo, Bergmann Ulrich (2003). The yeast mitochondrial proteome, a study of fermentative and respiratory growth.. J Biol Chem.

[PDF_01060] Orton R. J., Sellers W. I., Gerloff D. L. (2004). YETI: Yeast Exploration Tool Integrator.. Bioinformatics.

[PDF_00611] Palma Marco, DeLuca Darrow, Worgall Stefan, Quadri Luis E. N. (2004). Transcriptome analysis of the response of Pseudomonas aeruginosa to hydrogen peroxide.. J Bacteriol.

[PDF_00925] Perelman Susana, Mazzella María Agustina, Muschietti Jorge, Zhu Tong, Casal Jorge J. (2003). Finding unexpected patterns in microarray data.. Plant Physiol.

[PDF_00371] Poland Julia, Urbani Andrea, Lage Hermann, Schnölzer Martina, Sinha Pranav (2004). Study of the development of thermoresistance in human pancreatic carcinoma cell lines using proteome analysis.. Electrophoresis.

[PDF_00856] Pollet Nicolas, Delius Hajo, Niehrs Christof (2003). In situ analysis of gene expression in Xenopus embryos.. C R Biol.

[PDF_01064] Prlić Andreas, Domingues Francisco S., Lackner Peter, Sippl Manfred J. (2004). WILMA-automated annotation of protein sequences.. Bioinformatics.

[PDF_00731] Pyo Jaehoon, Hwang Sun-Il, Oh Jisun, Lee So-Jin, Kang Sun-Cheol, Kim Jong-Sang, Lim Jinkyu (2003). Characterization of a bovine pregnancy-associated protein using two-dimensional gel electrophoresis, N-terminal sequencing and mass spectrometry.. Proteomics.

[PDF_01062] Pérez A. J., Thode G., Trelles O. (2004). AnaGram: protein function assignment.. Bioinformatics.

[PDF_00378] Raza Shaan M., Fuller Gregory N., Rhee Chang Hun, Huang Suyun, Hess Kenneth, Zhang Wei, Sawaya Raymond (2004). Identification of necrosis-associated genes in glioblastoma by cDNA microarray analysis.. Clin Cancer Res.

[PDF_00858] Reinke Valerie, Gil Inigo San, Ward Samuel, Kazmer Keith (2003). Genome-wide germline-enriched and sex-biased expression profiles in Caenorhabditis elegans.. Development.

[PDF_01066] Reymond Nancie, Charles Hubert, Duret Laurent, Calevro Federica, Beslon Guillaume, Fayard Jean-Michel (2004). ROSO: optimizing oligonucleotide probes for microarrays.. Bioinformatics.

[PDF_01069] Rocca-Serra Philippe, Brazma Alvis, Parkinson Helen, Sarkans Ugis, Shojatalab Mohammadreza, Contrino Sergio, Vilo Jaak, Abeygunawardena Niran, Mukherjee Gaurab, Holloway Ele (2003). ArrayExpress: a public database of gene expression data at EBI.. C R Biol.

[PDF_00927] Rosati Barbara, Grau Frederic, Kuehler Anneke, Rodriguez Samantha, McKinnon David (2004). Comparison of different probe-level analysis techniques for oligonucleotide microarrays.. Biotechniques.

[PDF_00861] Rudolph Michael C., McManaman James L., Hunter Larry, Phang Tzulip, Neville Margaret C. (2003). Functional development of the mammary gland: use of expression profiling and trajectory clustering to reveal changes in gene expression during pregnancy, lactation, and involution.. J Mammary Gland Biol Neoplasia.

[PDF_00617] Sakabe Noboru Jo, de Souza Jorge E. S., Galante Pedro A. F., de Oliveira Paulo S. L., Passetti Fábio, Brentani Helena, Osório Elisson C., Zaiats André C., Leerkes Maarten R., Kitajima João Paulo (2003). ORESTES are enriched in rare exon usage variants affecting the encoded proteins.. C R Biol.

[PDF_00735] Sarry Jean-Emmanuel, Sommerer Nicolas, Sauvage François-Xavier, Bergoin Alexis, Rossignol Michel, Albagnac Guy, Romieu Charles (2004). Grape berry biochemistry revisited upon proteomic analysis of the mesocarp.. Proteomics.

[PDF_00383] Schaub Stefan, Rush David, Wilkins John, Gibson Ian W., Weiler Tracey, Sangster Kevin, Nicolle Lindsay, Karpinski Martin, Jeffery John, Nickerson Peter (2004). Proteomic-based detection of urine proteins associated with acute renal allograft rejection.. J Am Soc Nephrol.

[PDF_00621] Schmitt William A., Stephanopoulos Gregory (2003). Prediction of transcriptional profiles of Synechocystis PCC6803 by dynamic autoregressive modeling of DNA microarray data.. Biotechnol Bioeng.

[PDF_00930] Schramm A., Apostolov O., Sitek B., Pfeiffer K., Stühler K., Meyer H. E., Havers W., Eggert A. (2003). Proteomics: techniques and applications in cancer research.. Klin Padiatr.

[PDF_00389] Schwaenen Carsten, Nessling Michelle, Wessendorf Swen, Salvi Tatjana, Wrobel Gunnar, Radlwimmer Bernhard, Kestler Hans A., Haslinger Christian, Stilgenbauer Stephan, Döhner Hartmut (2004). Automated array-based genomic profiling in chronic lymphocytic leukemia: development of a clinical tool and discovery of recurrent genomic alterations.. Proc Natl Acad Sci U S A.

[PDF_00242] Schüller Christoph, Mamnun Yasmine M., Mollapour Mehdi, Krapf Gerd, Schuster Michael, Bauer Bettina E., Piper Peter W., Kuchler Karl (2003). Global phenotypic analysis and transcriptional profiling defines the weak acid stress response regulon in Saccharomyces cerevisiae.. Mol Biol Cell.

[PDF_01073] Segal Mark R., Dahlquist Kam D., Conklin Bruce R. (2003). Regression approaches for microarray data analysis.. J Comput Biol.

[PDF_00174] Sesterhenn Thomas M., Slaven Bradley E., Smulian A. George, Cushion Melanie T. (2003). Generation of sequencing libraries for the Pneumocystis Genome project.. J Eukaryot Microbiol.

[PDF_00525] Seta Karen A., Millhorn David E. (2004). Functional genomics approach to hypoxia signaling.. J Appl Physiol (1985).

[PDF_00145] Shatkay Hagit, Feldman Ronen (2003). Mining the biomedical literature in the genomic era: an overview.. J Comput Biol.

[PDF_00936] Shen Yufeng, Tolić Nikola, Masselon Christophe, Pasa-Tolić Ljiljana, Camp David G., Hixson Kim K., Zhao Rui, Anderson Gordon A., Smith Richard D. (2004). Ultrasensitive proteomics using high-efficiency on-line micro-SPE-nanoLC-nanoESI MS and MS/MS.. Anal Chem.

[PDF_01078] Shmueli Orit, Horn-Saban Shirley, Chalifa-Caspi Vered, Shmoish Michael, Ophir Ron, Benjamin-Rodrig Hila, Safran Marilyn, Domany Eytan, Lancet Doron (2003). GeneNote: whole genome expression profiles in normal human tissues.. C R Biol.

[PDF_00149] Simon Richard (2003). Using DNA microarrays for diagnostic and prognostic prediction.. Expert Rev Mol Diagn.

[PDF_00481] Sims Andrew H., Robson Geoffrey D., Hoyle David C., Oliver Stephen G., Turner Geoffrey, Prade Rolf A., Russell Hugh H., Dunn-Coleman Nigel S., Gent Manda E. (2004). Use of expressed sequence tag analysis and cDNA microarrays of the filamentous fungus Aspergillus nidulans.. Fungal Genet Biol.

[PDF_01082] Sjölander Kimmen (2004). Phylogenomic inference of protein molecular function: advances and challenges.. Bioinformatics.

[PDF_00151] Smith Clare V., Sacchettini James C. (2003). Mycobacterium tuberculosis: a model system for structural genomics.. Curr Opin Struct Biol.

[PDF_00395] Son Won-Kyu, Lee Do-Youn, Lee Sung-Han, Joo Won-A, Kim Chan-Wha (2003). Analysis of proteins expressed in rat plasma exposed to dioxin using 2-dimensional gel electrophoresis.. Proteomics.

[PDF_00153] Sparre Thomas, Bergholdt Regine, Nerup Jørn, Pociot Flemming (2003). Application of genomics and proteomics in Type 1 diabetes pathogenesis research.. Expert Rev Mol Diagn.

[PDF_00398] Sprenger Richard R., Speijer Dave, Back Jaap Willem, De Koster Chris G., Pannekoek Hans, Horrevoets Anton J. G. (2004). Comparative proteomics of human endothelial cell caveolae and rafts using two-dimensional gel electrophoresis and mass spectrometry.. Electrophoresis.

[PDF_00866] Srinivasan Prakash, Abraham Eappen G., Ghosh Anil K., Valenzuela Jesus, Ribeiro Jose M. C., Dimopoulos George, Kafatos Fotis C., Adams John H., Fujioka Hisashi, Jacobs-Lorena Marcelo (2003). Analysis of the Plasmodium and Anopheles transcriptomes during oocyst differentiation.. J Biol Chem.

[PDF_00940] Stanssens Patrick, Zabeau Marc, Meersseman Geert, Remes Gwen, Gansemans Yannick, Storm Niels, Hartmer Ralf, Honisch Christiane, Rodi Charles P., Böcker Sebastian (2004). High-throughput MALDI-TOF discovery of genomic sequence polymorphisms.. Genome Res.

[PDF_00402] Stevens E. V., Liotta L. A., Kohn E. C. (2003). Proteomic analysis for early detection of ovarian cancer: a realistic approach?. Int J Gynecol Cancer.

[PDF_00820] Strauss Arnold W. (2004). Tandem mass spectrometry in discovery of disorders of the metabolome.. J Clin Invest.

[PDF_00405] Strominger Jack L., Byrne Michael C., Wilson S. Brian (2003). Regulation of dendritic cell subsets by NKT cells.. C R Biol.

[PDF_00870] Strothmann Kai, Simoni Manuela, Mathur Premendu, Siakhamary Susan, Nieschlag Eberhard, Gromoll Jörg (2003). Gene expression profiling of mouse Sertoli cell lines.. Cell Tissue Res.

[PDF_00527] Stuitje Antoine R., Verbree Elizabeth C., van der Linden Karin H., Mietkiewska Elzbieta M., Nap Jan-Peter, Kneppers Tarcies J. A. (2003). Seed-expressed fluorescent proteins as versatile tools for easy (co)transformation and high-throughput functional genomics in Arabidopsis.. Plant Biotechnol J.

[PDF_00407] Swamy S. M. K., Tan P., Zhu Y. Z., Lu J., Achuth H. N., Moochhala S. (2004). Role of phenytoin in wound healing: microarray analysis of early transcriptional responses in human dermal fibroblasts.. Biochem Biophys Res Commun.

[PDF_00411] Takashima Motonari, Kuramitsu Yasuhiro, Yokoyama Yuuichiro, Iizuka Norio, Toda Toshifusa, Sakaida Isao, Okita Kiwamu, Oka Masaaki, Nakamura Kazuyuki (2003). Proteomic profiling of heat shock protein 70 family members as biomarkers for hepatitis C virus-related hepatocellular carcinoma.. Proteomics.

[PDF_00741] Tanaka N., Konishi H., Khan M. M. K., Komatsu S. (2003). Proteome analysis of rice tissues by two-dimensional electrophoresis: an approach to the investigation of gibberellin regulated proteins.. Mol Genet Genomics.

[PDF_00533] Tong Amy Hin Yan, Lesage Guillaume, Bader Gary D., Ding Huiming, Xu Hong, Xin Xiaofeng, Young James, Berriz Gabriel F., Brost Renee L., Chang Michael (2004). Global mapping of the yeast genetic interaction network.. Science.

[PDF_00415] Towbin Harry, Bair Kenneth W., DeCaprio James A., Eck Michael J., Kim Sunkyu, Kinder Frederick R., Morollo Anthony, Mueller Dieter R., Schindler Patrick, Song Hyun Kyu (2003). Proteomics-based target identification: bengamides as a new class of methionine aminopeptidase inhibitors.. J Biol Chem.

[PDF_00944] Tsai Chen-An, Hsueh Huey-miin, Chen James J. (2003). Estimation of false discovery rates in multiple testing: application to gene microarray data.. Biometrics.

[PDF_00629] Tsoi Stephen C. M., Cale Jacqueline M., Bird Ian M., Ewart Vanya, Brown Laura L., Douglas Susan (2003). Use of human cDNA microarrays for identification of differentially expressed genes in Atlantic salmon liver during Aeromonas salmonicida infection.. Mar Biotechnol (NY).

[PDF_00822] Tsoka Sophia, Ouzounis Christos A. (2003). Metabolic database systems for the analysis of genome-wide function.. Biotechnol Bioeng.

[PDF_00419] Tsubakihara Masako, Williams Neal K., Keogh Anne, dos Remedios Cristobal G. (2004). Comparison of gene expression between left atria and left ventricles from non-diseased humans.. Proteomics.

[PDF_00426] Unami Akira, Shinohara Yasuo, Kajimoto Kazuaki, Baba Yoshinobu (2004). Comparison of gene expression profiles between white and brown adipose tissues of rat by microarray analysis.. Biochem Pharmacol.

[PDF_00745] Ventelon-Debout Marjolaine, Delalande François, Brizard Jean-Paul, Diemer Hélène, Van Dorsselaer Alain, Brugidou Christophe (2004). Proteome analysis of cultivar-specific deregulations of Oryza sativa indica and O. sativa japonica cellular suspensions undergoing rice yellow mottle virus infection.. Proteomics.

[PDF_00750] Vierstraete Evy, Verleyen Peter, Baggerman Geert, D'Hertog Wannes, Van den Bergh Gert, Arckens Lutgarde, De Loof Arnold, Schoofs Liliane (2004). A proteomic approach for the analysis of instantly released wound and immune proteins in Drosophila melanogaster hemolymph.. Proc Natl Acad Sci U S A.

[PDF_00755] Wang Jingqiang, Xue Yanfen, Feng Xiaoli, Li Xiaolei, Wang Hao, Li Wei, Zhao Caifeng, Cheng Xiaojie, Ma Yanhe, Zhou Peijin (2004). An analysis of the proteomic profile for Thermoanaerobacter tengcongensis under optimal culture conditions.. Proteomics.

[PDF_01088] Wang Juhui, Caron Christophe, Mistou Michel-Yves, Gitton Christophe, Trubuil Alain (2004). PARIS: a proteomic analysis and resources indexation system.. Bioinformatics.

[PDF_00177] Wang Pauline W., Chu Linda, Guttman David S. (2004). Complete sequence and evolutionary genomic analysis of the Pseudomonas aeruginosa transposable bacteriophage D3112.. J Bacteriol.

[PDF_01091] Wang Song, Ethier Stewart (2004). A generalized likelihood ratio test to identify differentially expressed genes from microarray data.. Bioinformatics.

[PDF_00633] Watson Adam, Mata Juan, Bähler Jürg, Carr Anthony, Humphrey Tim (2003). Global gene expression responses of fission yeast to ionizing radiation.. Mol Biol Cell.

[PDF_00759] Weeks Mark E., James David C., Robinson Gary K., Smales C. Mark (2004). Global changes in gene expression observed at the transition from growth to stationary phase in Listeria monocytogenes ScottA batch culture.. Proteomics.

[PDF_00536] Wei Ho-Chun, Shu Huidy, Price James V. (2003). Functional genomic analysis of the 61D-61F region of the third chromosome of Drosophila melanogaster.. Genome.

[PDF_00433] Weigelt Britta, Glas Annuska M., Wessels Lodewyk F. A., Witteveen Anke T., Peterse Johannes L., van't Veer Laura J. (2003). Gene expression profiles of primary breast tumors maintained in distant metastases.. Proc Natl Acad Sci U S A.

[PDF_00158] Weinstein John N., Pommier Yves (2003). Transcriptomic analysis of the NCI-60 cancer cell lines.. C R Biol.

[PDF_00437] Wheelock Asa M., Zhang Lu, Tran Mai-Uyen, Morin Dexter, Penn Sharron, Buckpitt Alan R., Plopper Charles G. (2003). Isolation of rodent airway epithelial cell proteins facilitates in vivo proteomics studies of lung toxicity.. Am J Physiol Lung Cell Mol Physiol.

[PDF_00441] Whiteside Martin A., Chen Dung-Tsa, Desmond Renee A., Abdulkadir Sarki A., Johanning Gary L. (2004). A novel time-course cDNA microarray analysis method identifies genes associated with the development of cisplatin resistance.. Oncogene.

[PDF_01094] Wichert Sofia, Fokianos Konstantinos, Strimmer Korbinian (2004). Identifying periodically expressed transcripts in microarray time series data.. Bioinformatics.

[PDF_00225] Wiedenheft Blake, Stedman Kenneth, Roberto Francisco, Willits Deborah, Gleske Anne-Kathrin, Zoeller Luisa, Snyder Jamie, Douglas Trevor, Young Mark (2004). Comparative genomic analysis of hyperthermophilic archaeal Fuselloviridae viruses.. J Virol.

[PDF_00763] Wiemann Stefan, Mehrle Alexander, Bechtel Stephanie, Wellenreuther Ruth, Pepperkok Rainer, Poustka Annemarie, German cDNA Consortium (2003). CDNAs for functional genomics and proteomics: the German Consortium.. C R Biol.

[PDF_00445] Won Yonggwan, Song Ho-Jun, Kang Taek Won, Kim Jung-Ja, Han Byoung-Don, Lee Seung-Won (2003). Pattern analysis of serum proteome distinguishes renal cell carcinoma from other urologic diseases and healthy persons.. Proteomics.

[PDF_01097] Wren Jonathan D., Garner Harold R. (2004). Shared relationship analysis: ranking set cohesion and commonalities within a literature-derived relationship network.. Bioinformatics.

[PDF_00448] Xiao Zhen, Conrads Thomas P., Lucas David A., Janini George M., Schaefer Carl F., Buetow Kenneth H., Issaq Haleem J., Veenstra Timothy D. (2004). Direct ampholyte-free liquid-phase isoelectric peptide focusing: application to the human serum proteome.. Electrophoresis.

[PDF_00488] Yamashita Riu, Suzuki Yutaka, Nakai Kenta, Sugano Sumio (2003). Small open reading frames in 5' untranslated regions of mRnas.. C R Biol.

[PDF_00766] Yoshimura Yoshiyuki, Yamauchi Yoshio, Shinkawa Takashi, Taoka Masato, Donai Hitomi, Takahashi Nobuhiro, Isobe Toshiaki, Yamauchi Takashi (2004). Molecular constituents of the postsynaptic density fraction revealed by proteomic analysis using multidimensional liquid chromatography-tandem mass spectrometry.. J Neurochem.

[PDF_00771] Yu Ji Hoon, Yun Shin Young, Lim Joo Weon, Kim Hyeyoung, Kim Kyung Hwan (2003). Mass spectrometry and tandem mass spectrometry analysis of rat mitochondrial ATP synthase: up-regulation in pancreatic acinar cells treated with cerulein.. Proteomics.

[PDF_00452] Yu Ji Hoon, Yun Shin Young, Lim Joo Weon, Kim Hyeyoung, Kim Kyung Hwan (2003). Proteome analysis of rat pancreatic acinar cells: implication for cerulein-induced acute pancreatitis.. Proteomics.

[PDF_00775] Zhang Bo, Su Yong-Ping, Ai Guo-Ping, Liu Xiao-Hong, Wang Feng-Chao, Cheng Tian-Min (2003). Differentially expressed proteins of gamma-ray irradiated mouse intestinal epithelial cells by two-dimensional electrophoresis and MALDI-TOF mass spectrometry.. World J Gastroenterol.

[PDF_00163] Zhang Hong-Gang, Xiu Rui-Juan (2003). Micro-vascular medicine and proteomics.. Clin Hemorheol Microcirc.

[PDF_00455] Zhang Rulin, Barker Lisa, Pinchev Deborah, Marshall John, Rasamoelisolo Michèle, Smith Chris, Kupchak Peter, Kireeva Inga, Ingratta Leslee, Jackowski George (2004). Mining biomarkers in human sera using proteomic tools.. Proteomics.

[PDF_01102] Zhang Yu, Waterman Michael S. (2003). An Eulerian path approach to global multiple alignment for DNA sequences.. J Comput Biol.

[PDF_00882] Zhao Po, Seo Jinwook, Wang Zuyi, Wang Yue, Shneiderman Ben, Hoffman Eric P. (2003). In vivo filtering of in vitro expression data reveals MyoD targets.. C R Biol.

[PDF_00959] Zheng Lianxing, Liu Jun, Batalov Sergei, Zhou Demin, Orth Anthony, Ding Sheng, Schultz Peter G. (2003). An approach to genomewide screens of expressed small interfering RNAs in mammalian cells.. Proc Natl Acad Sci U S A.

[PDF_01104] Zhu Qingsong, Deng Youping, Vanka Phaneendra, Brown Susan J., Muthukrishnan Subbaratnam, Kramer Karl J. (2004). Computational identification of novel chitinase-like proteins in the Drosophila melanogaster genome.. Bioinformatics.

[PDF_00180] Zimdahl Heike, Nyakatura Gerald, Brandt Petra, Schulz Herbert, Hummel Oliver, Fartmann Berthold, Brett David, Droege Marcus, Monti Jan, Lee Young-Ae (2004). A SNP map of the rat genome generated from cDNA sequences.. Science.

[PDF_00202] von Mering Christian, Zdobnov Evgeny M., Tsoka Sophia, Ciccarelli Francesca D., Pereira-Leal Jose B., Ouzounis Christos A., Bork Peer (2003). Genome evolution reveals biochemical networks and functional modules.. Proc Natl Acad Sci U S A.

